# Overview on the Sensing Materials and Methods Based on Reversible Addition–Fragmentation Chain-Transfer Polymerization

**DOI:** 10.3390/bios15100673

**Published:** 2025-10-07

**Authors:** Zhao-Jiang Yu, Lin Liu, Su-Ling Yang, Shuai-Bing Yu

**Affiliations:** 1 College of Chemistry and Chemical Engineering, Anyang Normal University, Anyang 455000, China; yzj86jiang@aynu.edu.cn (Z.-J.Y.); yang-sl@aynu.edu.cn (S.-L.Y.); 2 College of Chemical and Environmental Engineering, Anyang Institute of Technology, Anyang 455000, China

**Keywords:** reversible addition–fragmentation chain-transfer, electrochemical sensors, optical sensors, signal amplification

## Abstract

Reversible addition–fragmentation chain-transfer (RAFT) polymerization has become an efficient method in the field of polymer synthesis. Recently, the RAFT polymerization technique has been successfully used to prepare functional materials and develop various sensing methods used in different scenarios. The polymerization reaction can be initiated by thermal, electrochemical, photochemical, enzymatic, and mechanical stimulation. More interestingly, RAFT polymerization can be performed in situ by recruiting a large number of signal tags at the solid interface to amplify the signals. In this review, we addressed the latest achievements in the preparation of sensing materials and the design of different sensors based on the RAFT polymerization technique for sensing ions and small molecules and bioimaging of tumor cells and viruses. Then, electrochemical and optical biosensors through the signal amplification of the RAFT polymerization method were summarized. This work could provide inspiration for researchers to prepare fascinating sensing materials and develop novel detection technologies applied in various fields.

## 1. Introduction

With the rapid development of material science and analytical technology, the sensing field has been committed to improving the sensitivity, selectivity, and stability of detection methods to meet the needs of different scenarios. The development of high-performance sensors is crucial for the precise detection of disease biomarkers in biomedical applications and trace pollutants in environmental monitoring [1]. Among the factors that may affect sensor performance, the construction and modification of sensing materials play a central role. Reversible addition–fragmentation chain-transfer (RAFT) polymerization, a key member of the controlled radical polymerization family, has rapidly emerged in the field of polymer synthesis since it was proposed by Rizzardo’s team in 1998 [2]. In traditional free radical polymerization systems, a high concentration of free radicals can lead to the termination of the polymerization reaction, resulting in a wide molecular weight distribution of polymers, making it difficult to accurately adjust the polymer structure and property. In contrast, RAFT polymerization can induce the degradation transfer between growing free radicals and chain transfer agents (CTAs) with high chain transfer constants, such as bis (thio) ester derivatives, to reduce the concentration of free radicals, thereby enabling precise control of the polymerization reaction [3]. In addition, RAFT polymerization has a wide range of monomers, eliminating the need for expensive reagents and avoiding the inherent problem of difficult impurity removal in the reaction of atom transfer radical polymerization [4]. Over the years, the RAFT polymerization technique has been used to develop various sensing methods used in different scenarios and has become a basic technique in laboratory and industrial production because it is easy to operate and does not require additional complex equipment [5]. In the sensing field, RAFT polymerization has attracted widespread attention due to its ability to precisely regulate polymer structure and property. In the construction of sensors, RAFT polymerization plays a role mainly in the following two ways: (1) functional polymers with specific recognition groups to achieve selective recognition of target analytes can be synthesized through RAFT polymerization technique [6,7,8,9]. For example, polymers containing antigens/antibodies, aptamers, molecular imprints, and other recognition units have been prepared by RAFT polymerization to specifically capture pollutants and disease markers, significantly improving the selectivity and sensitivity of sensors. (2) Signal amplification can be achieved by RAFT polymerization by introducing a large number of signal probes (e.g., fluorescent dyes and electroactive tags) into the polymer chains through the chain growth characteristics.

RAFT polymerization has sparked great research enthusiasm worldwide since its first report in 1998. Recently, some interesting review papers related to RAFT polymerization have been reported. However, these reviews mainly focus on or involve the RAFT-assisted synthesis of functional materials and the environmental and biomedical applications of RAFT polymerization. For example, Nothling et al. and Lee et al. have addressed the progress and perspectives of traditional and photocontrolled RAFT polymerization [3,10]; the groups of Keddie, Vana, and Boyer have summarized the design and synthesis of block copolymers and functional materials using the RAFT polymerization technique [11,12,13], and the groups of Davis, Dhara, and Whittaker have introduced RAFT polymerization-derived organic/inorganic nanomaterials for biomedical applications [14,15,16]. Although limited chapters have involved RAFT polymerization-based sensors in the early reviews [17,18,19], there is no specific review to systematically address their design and sensing applications. Therefore, given the unique advantages of RAFT polymerization in the precise preparation of materials and rapid development of sensors, it is necessary to systematically summarize the latest achievements of RAFT-based sensing methods and applications. This article mainly summarized the applications of the RAFT polymerization technique in the preparation of polymers or polymeric materials for sensing ions and small molecules and bioimaging of tumor cells and viruses, as well as the signal amplification for the design of electrochemical and optical biosensors. Finally, we conclude with an outlook on future developments, aiming to provide inspiration for relevant researchers. We did not provide a detailed introduction to the structure feature and synthesis method of RAFT polymers, as interested researchers can read the impressive review papers to find the solutions [10,12,20,21].

## 2. RAFT Polymerization: Mechanisms and Advantages

**Scheme 1:**
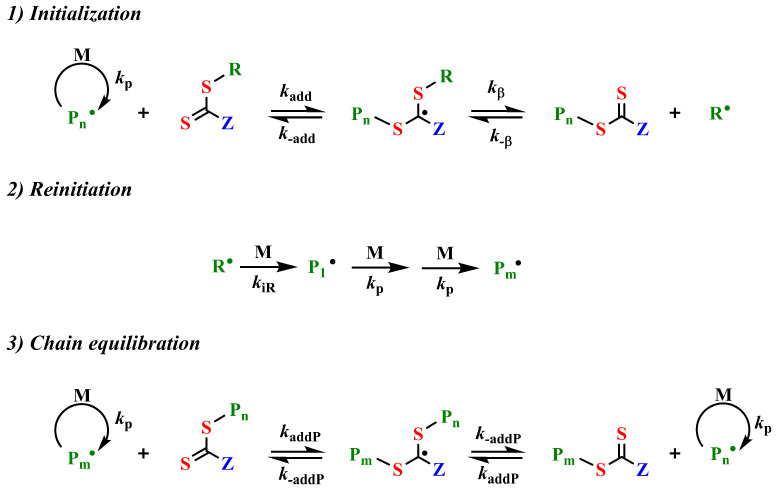
The mechanism of RAFT polymerization.

As a key technology for living/controlled radical polymerization, RAFT polymerization can achieve precise polymerization regulation through the core mechanism predicated on the “reversible addition–fragmentation” reaction between the dithioester-based RAFT agent and active radical [17]. As depicted in Scheme 1, the primary radical generated from initiator decomposition first reacts with the monomer to form an active propagating chain, which subsequently undergoes an addition reaction with the C=S moiety of CTA, resulting in the formation of an unstable intermediate radical. This intermediate promptly undergoes cleavage, producing two radical species: an active radical carrying a RAFT fragment, which can sustainably initiate monomer polymerization, and an R-group radical, which can initiate new chain growth. The dynamic equilibrium established between these two species effectively reduces the random termination of free radicals, thereby ensuring the uniform propagation of polymer chains. In this process, sulfur-containing CTA Z−C−(=S)S−R can form a dormant intermediate Z−C−(=S)S−P_n_ with growth radical P_n_**^•^**, which can greatly inhibit the irreversible double radical termination side reaction between the growth radicals. The dormant intermediate cleaves itself to release a new reactive radical R**^•^** from the sulfur atom, which can bind with the monomer to maintain the growth chain. The rapid transfer of CTA between active and dormant radicals can lead to a significant narrowing of molecular weight distribution, making the polymerization reaction controllable.

**Scheme 2:**
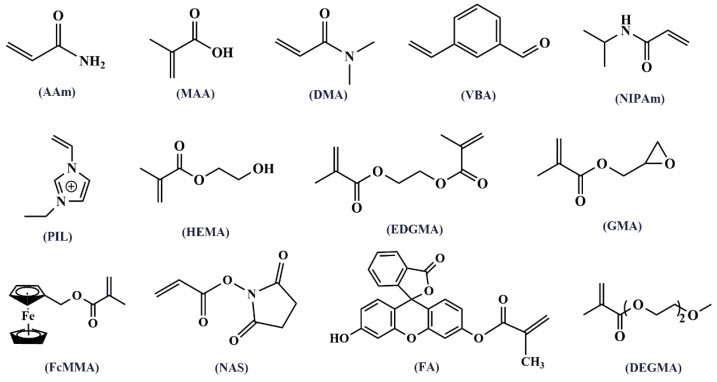
The structures of different types of monomers used for the preparation of sensing materials: AAm, acrylamide; MAA, methylacrylic acid; DMA, N,N-dimethylacrylamide; VBA, 3-vinylbenzaldehyde; NIPAm, N-isopropyl acrylamide; PIL, polymer ionic liquid; HEMA, 2-hydroxyethyl methacrylate; EDGMA, ethylene dimethacrylate; GMA, glycidyl methacrylate; FcMMA, ferrocenylmethyl methacrylate; NAS, N-acryloxysuccinimide; FA, fluorescein O-methacrylate; DEGMA, di(ethylene glycol) methyl ether methacrylate.

Compared to other controlled radical polymerization methods such as atom transfer radical polymerization (ATRP), nitroxide mediated polymerization (NMP), and single electron transfer living radical polymerization (SET-LRP) [3,12], RAFT polymerization exhibits significant advantages: (1) it is applicable to a wide range of monomers (Scheme 2), (2) the reaction conditions are generally mild (typically conducted at room temperature and atmospheric pressure, and compatible with various polymerization modes such as solution and emulsion polymerization), and (3) it enables precise control over polymer molecular weight, end-group functionalization, and topology (e.g., block copolymers, star copolymers, etc.). Additionally, this method is free of metal residues and exhibits high tolerance to impurities, making it particularly suitable for the synthesis of biomedical materials and functional polymers, with broad prospects in material science and biomedical applications. Among kinds of polymerization techniques, RAFT and ATRP are two of the most common controlled radical polymerization methods. Both techniques can produce functional polymers with predetermined length, composition, dispersity, and end groups. The fundamental differences and similarities between the two methods are shown in Table 1 [4]. To overcome the limitations of an independent living radical polymerization method, one possible approach is to integrate RAFT with other polymerization techniques together [22].

## 3. Polymer Architecture Design for Sensing

The core value of RAFT polymerization technology in the sensing field has primarily stemmed from its ability to precisely regulate the molecular chain structure, functional group distribution, and topological morphology of resulting polymers. By designing active functional monomers containing carboxyl and amino groups, RAFT polymerization technology enables the preparation of linear or block polymers with excellent biocompatibility. On the one hand, such polymers allow for the immobilization of biological recognition molecules such as antibodies and nucleic acids on the sensing interface through covalent bonding or specific interactions, ensuring the effective retention of recognition elements [23,24]. On the other hand, the polymers can effectively prevent the non-specific adsorption of impurity molecules due to their hydrophilicity and steric hindrance effect. On this basis, RAFT polymerization technology further breaks through the functional boundaries of materials. The synthesized conductive polymers or polymeric nanocomposites loaded with fluorescent groups are endowed with a dual core function of “recognition-signal conversion”. When being used as signal probes, the optical properties (e.g., fluorescence intensity, ultraviolet absorption) of these materials will change upon binding to specific metal ions or small molecules, which enables the qualitative and quantitative detection of targets. When being used as electrode modification materials, the good conductivity of polymers can efficiently convert the interaction between the target and the electrode surface into a quantifiable electrochemical signal, significantly expanding the detection range and improving the sensing sensitivity. In the scenario of bioimaging, the polymers synthesized by RAFT polymerization technology can accurately recognize and bind to the targets on the cell surface, achieving high-resolution cell imaging in combination with fluorescent signal units. Therefore, RAFT polymerization technology has fully completed the advantageous upgrade from “precision synthesis of functional materials” to “multi-scenario sensing applications”.

The different initiation modes of RAFT polymerization exhibit distinct characteristics for practical applications. Compared with ATRP, thermally initiated RAFT polymerization is beneficial for sensitive biological detection because it can prevent interference from non-specific binding during the signal collection and has the advantages of low cost, high efficiency, and relatively simple operation [25]. However, the rapid and effective initiation of thermally initiated RAFT requires a high-temperature anaerobic environment, which may lead to denaturation or inactivation of biomolecules such as nucleic acids and proteins. Unlike thermally initiated RAFT, electrochemically mediated RAFT (eRAFT) can be conducted under relatively mild conditions. In eRAFT, diazonium salts are greatly reduced, controlling the generation of free radicals without damaging the CTAs [26]. For this view, the mild reaction conditions of eRAFT significantly improve the convenience of biomolecular detection platforms. Although biosensors based on eRAFT polymerization can solve the problem of deoxygenation, there are still some limitations, such as cumbersome reaction setups and requirements for sequential polymerization reactions, which are unfavorable for practical applications and will bring significant time costs. Photo-induced electron transfer–RAFT (PET-RAFT) polymerization can effectively avoid damage to heat-sensitive monomers or functional groups and reduce environmental pollution due to its mild reaction conditions. It meets the requirements of green chemistry and biocompatibility, thus showing unique advantages in the field of biosensing [10]. In addition, photoinitiated polymerization offers excellent spatiotemporal control, allowing for precise regulation of reaction initiation and termination through light on/off and enabling spatial localization with optical devices to precisely control polymer growth at the microscale. However, PET-RAFT reactions require precise light source control equipment, which will increase the detection cost and operational threshold and thus is unfavorable for rapid promotion in primary healthcare settings.

## 4. Target-Specific Sensing Applications

### 4.1. Polymeric Materials Prepared by RAFT Polymerization for Sensing of Ions

**Figure 1:**
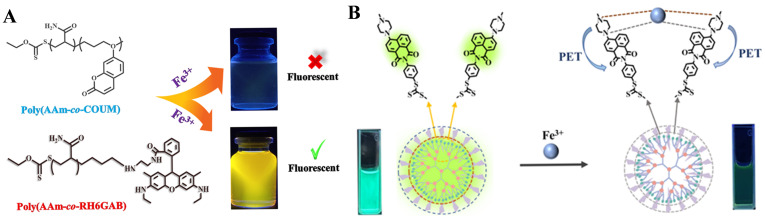
(**A**) Schematic illustration of fluorescent polymer probe for Fe^3+^ detection. Reprinted with permission from ref. [31]. Copyright 2023 Elsevier B.V. (**B**) Schematic illustration of fluorescent detection mechanism for Fe^3+^ with FL-AHBP-1. Reprinted with permission from ref. [32]. Copyright 2024 Elsevier B.V.

Ions exist throughout our environments, from living organisms to agricultural products and other fields. Sensing and monitoring of ions have been research hotspots since their pollution is harmful to human health. Among various methods and materials for ion sensing, polymer-based probes have the advantages of high sensitivity, selectivity, and stability [27]. Polymers consisting of conjugated backbones and multifunctional side chains have been prepared by RAFT polymerization and used for the separation and sensing of different ions (Table 2), including metal ions (e.g., iron, aluminum, copper, mercury, chromium, and zinc) and anion ions [28,29,30,31,32,33,34,35,36,37,38,39,40,41,42,43,44,45,46,47]. Iron ions maintain the normal function of organs and life metabolic processes, but their abnormal content in the human body is associated with many diseases. Fluorescent polymeric probes prepared by the RAFT polymerization technique have been used for sensing iron ions based on the specific metal coordination interactions with the binding moieties in the polymers. For example, Gheitarani et al. prepared several fluorescent polymers through RAFT polymerization of acrylamide (AAm) monomers with N-(rhodamine-6G) lactam-N′-allyl-ethylenediamine (RH6GAB) and 7-(allyloxy) 2H-chromen-2-one (AC) as fluorescent chromophores. It was found that coumarin- and rhodamine-based copolymer P(AAm-*co*-AC)-*b*-(AAm-*co*-RH6GAB) could be used for the selective detection of Fe^3+^ by a ligand–metal charge transfer (LMCT) mechanism (Figure 1A) [31]. Zhao et al. synthesized an amphiphilic hyper-branched fluorescent polymer (FL-AHBP-1) for Fe^3+^ determination with a multifunctional RAFT reagent containing fluorophore naphthalene, dithionate, and a C=C double bond (Figure 1B) [32]. Through the photoinduced electron/charge transfer (PET) mechanism, the fluorescence quenching efficiency of Fe^3+^ toward FL-AHBP-1 reached 74.4%.

**Figure 2:**
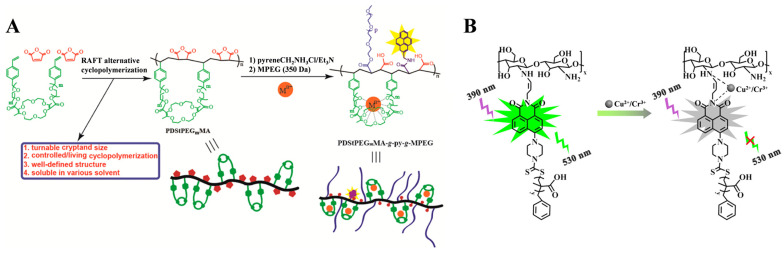
(**A**) Schematic illustration for synthesis of pseudo-cryptand-containing copolymers by alternative RAFT cyclocopolymerization. Reprinted with permission from ref. [36]. Copyright 2016 American Chemical Society. (**B**) Schematic illustration of possible recognition mechanisms for Cr^3+^ and Cu^2+^. Reprinted with permission from ref. [42]. Copyright 2024 Elsevier B.V.

In addition to the polymerization of small RAFT monomers, macromolecules and natural polymers such as poly(ethylene glycol), cellulose, and chitosan can be grafted onto the polymeric backbones to improve the water solubility and biocompatibility of copolymers. For example, Zhang et al. synthesized pseudo-cryptand-containing copolymers (PDStPEGmMA) with different sizes by maleic anhydride (MAn)-based RAFT polymerization of distyrenic 18-membered macrocycle derivatives (Figure 2A) [36]. Pyrene and hydrophilic methoxypoly(ethylene glycol) (MPEG) tags were grafted onto the polymeric backbones to endow the polymers with strong fluorescence and good water solubility. The fluorescent PDStPEG_2_MA_10_-*g*-py-*g*-MPEG could be used for selective sensing of Al^3+^ ions. Chitosan is a plentiful and easily available natural polymer. Chen et al. prepared naphthalimide-based fluorescent copolymers with methylacrylic acid (MAA) as the RAFT monomers by introducing chitosan in the polymer side chains (Figure 2B) [42]. The fluorescent copolymers could be used for the determination of Cr^3+^ and Cu^2+^ with a detection limit of 44.6 nM and 54.5 nM, respectively.

**Figure 3:**
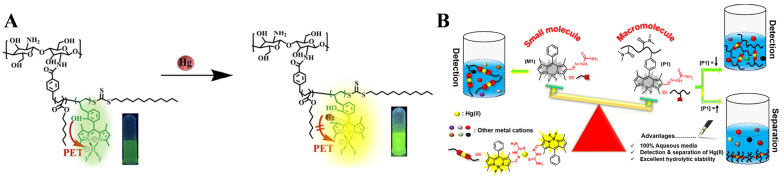
(**A**) Schematic illustration for possible Hg(II)-binding mechanism of chitosan-*g*-PHMA-BDP fluorescent sensor. Reprinted with permission from ref. [43]. Copyright 2023 Elsevier B.V. (**B**) Schematic representation of the Hg(II) detection and separation. Reprinted with permission from ref. [44]. Copyright 2019 American Chemical Society.

The accumulation of mercury in the human body, even in the range of a few parts per million, can cause serious health consequences. Due to the rapid development of industrialization and urbanization, the pollution level of mercury ions in waterways has been steadily increasing. Fluorescent polymers prepared by RAFT polymerization have been reported for the detection of Hg(II)/Hg(I) ions. For example, He et al. prepared several chitosan-based fluorescent co-polymers by RAFT polymerization and found that chitosan-*g*-polyhexyl methacrylate-allyl boron-dipyrrolemethene (chitosan-*g*-PHMA-BDP) showed the highest sensitivity for selective recognition of Hg^2+^/Hg^+^ ions by PET effect (Figure 3A) [43]. The polymers could be easily processed into films and coatings, promoting the development of portable devices for Hg(II)/Hg(I) sensing. Haldar et al. reported a water-soluble BODIPY-derived polymer for simultaneous detection and separation of Hg(II) ions (Figure 3B) [44]. The polymer scaffold poly(N,N′-dimethyl acrylamide-*co*-5,5-difluoro-1,3,7,9-tetramethyl-10-phenyl-5H-dipyrrolo-iazaborinine-2-carbaldehyde) was synthesized by RAFT polymerization and then modified with thiosemicarbazide. The polymer probe showed bright yellow emission in the presence of Hg(II) ions. Selective removal of Hg(II) ions could be achieved by precipitation of the polymer-Hg(II) complexes.

**Figure 4:**
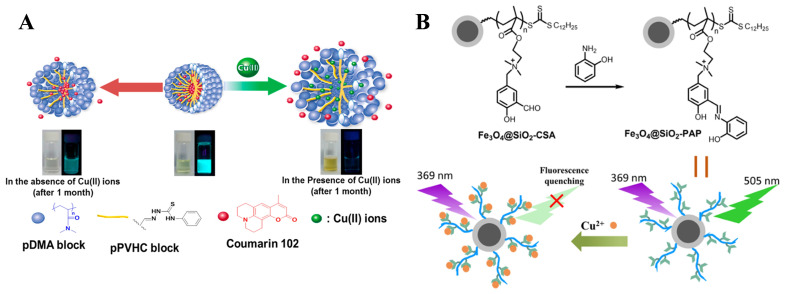
(**A**) Schematic illustration for encapsulated dyes within the hydrophobic core of p(DMA-*b*-PVHC) micelles right after micellization (**middle**), after 1 month in the absence of Cu(II) ions (**left**), and the complete release of encapsulated dyes after 1 month in the presence of Cu(II) ions (**right**). Reprinted with permission from ref. [48]. Copyright 2019 American Chemical Society. (**B**) Schematic illustration for fluorescence quenching of multifunctional nanohybrid Fe_3_O_4_@SiO_2_-PAP. Reprinted with permission from ref. [45]. Copyright 2023 American Chemical Society.

In addition to fluorescent sensing and removing Hg(II) ions, RAFT-based copolymers and nanohybrids have been prepared and used for detecting/removing other metal ions and monitoring metal ion-triggered drug release. For example, Bak et al. reported an amphiphilic phenylthiosemicarbazone-based block copolymer for determining Cu^2+^ and monitoring Cu^2+^-triggered drug release (Figure 4A) [48]. Poly(N,N-dimethylacrylamide) (pDMA) synthesized by RAFT polymerization of N,N-dimethylacrylamide (DMA) was modified with 3-vinylbenzaldehyde (VBA) to form poly[(N,N-dimethylacrylamide)-*b*-(3-vinylbenzaldehyde)] [p(DMA-*b*-VBA)]. Then, p(DMA-*b*-VBA) was modified with phenylthiosemicarbazide to yield poly{N,N-dimethylacrylamide-*b*-[N-phenyl 2-(3-vinylbenzylidene)hydrazine carbothioamide]} [p(DMA-*b*-PVHC)]. The block copolymers p(DMA-*b*-PVHC) can self-assemble into polymeric micelles in an aqueous solution. The coordination interaction between Cu(II) ions and phenylthiosemicarbazone units in the micelles caused a color change from colorless to yellow. After slow penetration of Cu(II) ions, hydrophobic coumarin drugs encapsulated in the micelles were released due to the gradual swelling of the cross-linked micelle cores. In addition, Zhang et al. reported a nanohybrid probe for sensing and removing Cu^2+^ by grafting a quaternized salicylaldehyde Schiff base side-chain polymer on the silica-coated Fe_3_O_4_ nanoparticles (Fe_3_O_4_@SiO_2_-PAP) (Figure 4B) [45]. The nanohybrid was prepared by RAFT polymerization of 2-(dimethylamino)ethyl methacrylate (DMAEMA) and subsequent quaternization of 5-chloromethylsalicylaldehyde for Schiff condensation with 2-aminophenol. The fluorescence of Fe_3_O_4_@SiO_2_-PAP could be effectively quenched by Cu^2+^ ions by a static quenching mechanism.

**Figure 5:**
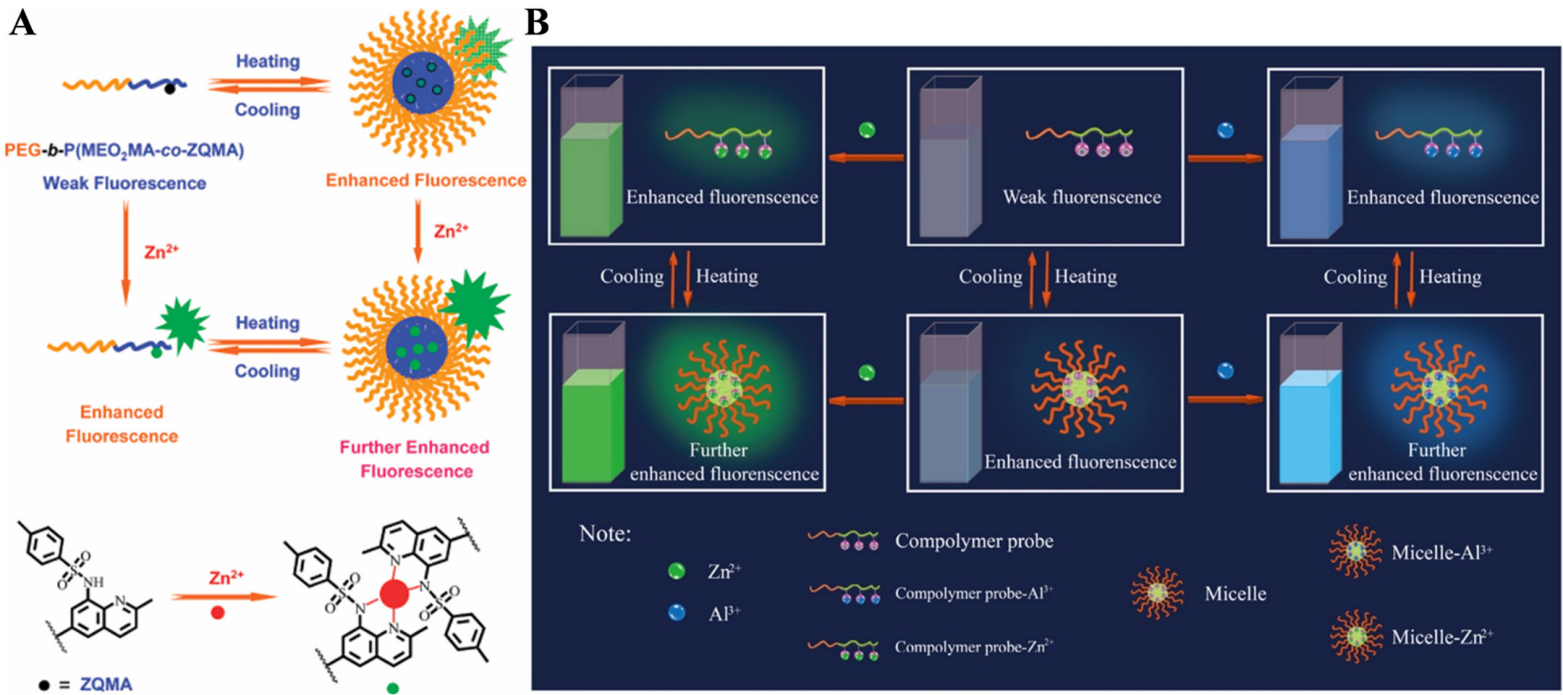
(**A**) Schematic illustration for the fabrication of thermoresponsive PEG-*b*-P(MEO_2_MA-*co*-ZQMA) double hydrophilic block copolymer-based fluorescent chemosensors of Zn^2+^ ions and temperature. Reprinted with permission from ref. [46]. Copyright 2011 American Chemical Society. (**B**) Schematic illustration for the fabrication of thermoresponsive PEO_113_-*b*-P(NIPAM-*co*-SHMA)_65_ and responsive sensing of Al^3+^, Zn^2+^ and temperature. Reprinted with permission from ref. [47]. Copyright 2017 Elsevier B.V.

Stimulus-responsive block copolymers that exhibit reversible or irreversible changes in physical properties or chemical structures have attracted increasing attention. They can provide a range of potential features, such as improved water solubility, enhanced detection sensitivity, and excellent biocompatibility. Liu et al. fabricated stimulus-responsive double hydrophilic block copolymers with quinoline-based Zn^2+^-recognizing moieties (ZQMA) for fluorescent sensing of Zn^2+^ ions and temperature (Figure 5A) [46]. PEG-*b*-P(MEO_2_MA-*co*-OEGMA-*co*-ZQMA) was synthesized by RAFT polymerization of 2-(2-methoxyethoxy)ethyl methacrylate (MEO_2_MA), oligo(ethylene glycol) monomethyl ether methacrylate (OEGMA), and ZQMA in the presence of a PEG-based macroRAFT agent. Binding of PEG-*b*-P(MEO_2_MA-*co*-ZQMA) polymer with Zn^2+^ ions led to an increase in fluorescence intensity. The PEG-*b*-P(MEO_2_MA-*co*-ZQMA) polymers self-assembled into micelles upon heating, leading to further enhancement in the fluorescence intensity after the addition of Zn^2+^. The in vitro studies indicated that the micelles could enter into living cells for fluorescence imaging of Zn^2+^ ions. This work represented a new Zn^2+^-sensing system by integrating a Zn^2+^-binding moiety with the stimuli-responsive copolymer. In addition, Han et al. reported responsive double hydrophilic block copolymers for sensing Al^3+^, Zn^2+^, and temperature (Figure 5B) [47]. The Al^3+^- and Zn^2+^-binding thermosensitive block poly(ethylene oxide)-*b*-poly(N-isopropylacrylamide-*co*-SHMA)_65_ (PEO_113_-*b*-P(NIPAM-*co*-SHMA)_65_ copolymers were prepared by RAFT polymerization. Binding of the copolymers with Al^3+^ and Zn^2+^ led to blue and green fluorescence, respectively. The detection limit decreased from 5.23 to 2.14 ppb for Al^3+^ and from 11.99 to 8.71 ppb for Zn^2+^ when the PEO_113_-*b*-P(NIPAM-*co*-SHMA) polymers self-assembled into micelles under heating.

**Figure 6:**
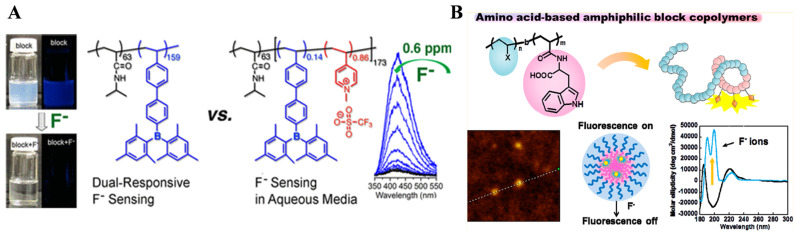
(**A**) Schematic illustration of electron-deficient triarylborane block copolymers for F^−^ sensing. Reprinted with permission from ref. [52]. Copyright 2013 American Chemical Society. (**B**) Schematic illustration of tryptophan-containing block copolymers for F^−^ sensing. Reprinted with permission from ref. [53]. Copyright 2013 American Chemical Society.

In addition to metal ions, anion sensing is also one of the most active areas of supramolecular chemistry, especially for the detection of environmentally deleterious and potentially toxic anions [49,50,51]. Fluoride has become a general research target for anion sensing due to its importance in biological and medical processes. Cheng et al. synthesized an electron-deficient triarylborane block copolymer by RAFT polymerization of vinyl-functionalized borane monomers (Figure 6A) [52]. The block copolymer served as a dual-responsive probe for F^−^ sensing in a polar solvent. The distribution of the borane chromophore along the polymer chain had a significant impact on the F^−^ binding response. The positive charge on the polymer chain enhanced F^−^ binding strength, allowing it to be recognized in a highly polar solvent and even in an aqueous solution. In addition, Mori et al. reported a tryptophan-based block RAFT copolymer for F^−^ sensing (Figure 6B) [53]. Tryptophan-containing acrylamide, *N*-acryloyl-L-tryptophan (A-Trp-OH), was used as the monomer for RAFT polymerization. The polymer could self-assemble into spherical micelles consisting of a hydrophobic core of poly(A-Trp-OH) and a hydrophilic shell, further enhancing ratiometric fluorescent sensing capability for F^−^ in aqueous and organic media.

Colorimetric sensors for sensing ions have been widely reported in studies. Different carriers, including papers, hydrogels, silica particles, and polymeric films, can be employed as substrates for immobilizing ion receptors. Among them, polymeric films are particularly promising due to their advantages of cost-effectiveness, flexibility, and stability [48,54,55,56,57]. For this view, Li et al. prepared a water-soluble polymer with plenty of rhodamine B dyes by RAFT polymerization [54]. The polymer could be used to detect Cu^2+^ ions based on the color change from colorless to purple. In addition, Huang et al. reported a paper-based sensor for colorimetric sensing of Cd^2+^ ions using RAFT-based imprinted polymers [55]. Phillips et al. reported a gold nanoparticles (AuNPs)-based colorimetric sensing system for Fe^3+^ detection by modifying catechol groups on the surface of RAFT-polymeric nanoparticles [56].

**Table 2:** Analytical performances of RAFT-based materials for ion sensing.

Material	Ion	Linear Range	Detection Limit	Ref.
BODIPY-derived polymer	Fe^3+^	1–15 μM	1.2 μM	[30]
Naphthalene-derived polymer	Fe^3+^	0–15 μM	1.82 nM	[32]
Azo-Schiff base polymer	Fe^3+^	0.1–1.3 mM	0.1 mM	[33]
NBN-derived polymer	Fe^3+^, Cr^3+^	1–6.5 μM, 0–10 μM	7.3 nM, 14.69 nM	[34]
Coumarin-derived β-CD	Fe^3+^	0–16 μM	0.34 μM	[35]
Pyrene-derived polymer	Al^3+^	0–0.6 μM	0.22 μM	[36]
Salicylaldehyde-derived polymer	Al^3+^, Zn^2+^	–	2.14 ppb, 8.71 ppb	[47]
Benzaldehyde and Rh6G-derived polymer	Al^3+^, Fe^3+^	–	30 nM, 5.95 nM	[37]
Quinoline based polymer	Zn^2+^	–	3 nM	[46]
BODIPY-derived polymer	Hg^2+^	0–20 µM	1.1 µM	[39]
BODIPY-derived polymer	Hg^2+^	0–2 μM	0.37 μM	[44]
BODIPY-derived chitosan	Hg^2+^, Hg^+^	0–20 μM	0.61 μM, 0.47 μM	[43]
Fe_3_O_4_@SiO_2_-PAP	Cu^2+^	0.1–2 µg/mL	0.125 µM	[45]
GO-LP/PMAM	Cu^2+^	0.25–2 mM	0.19 mM	[40]
Polymeric micelles	Cu^2+^	33–100 μM	–	[48]
Naphthalimide-derived chitosan	Cr^3+^, Cu^2+^	0–10 μM	44.6 nM, 54.5 nM	[42]
ZnO QDs	Cr^6+^	–	1.13 µM	[41]
Colorimetric polymer probe	Cu^2+^	–	0.18 nM	[54]
Ion imprinted polymer paper	Cd^2+^	1–100 ng/mL	0.4 ng/mL	[55]
Polymer-AuNPs	Fe^3+^	8–25 mM	–	[56]
Hemicyanine-based probe	CN^−^	7–140 μM	2.24 μM	[57]

Abbreviations: BODIPY, boron dipyrromethene; NBN, nitrogen–boron–nitrogen; β-CD, β-cyclodextrin; Fe_3_O_4_@SiO_2_-PAP, quaternized salicylaldehyde Schiff base side-chain polymer grafted magnetic Fe_3_O_4_ nanoparticles; GO, graphene oxide; LP, L-phenylalanine; PMAM, polymethacrylamide; QDs, quantum dots; AuNPs, gold nanoparticles.

### 4.2. Polymeric Materials Prepared by RAFT Polymerization for Sensing of Small Molecules

#### 4.2.1. Electrochemical Sensing of Small Molecules

**Figure 7:**
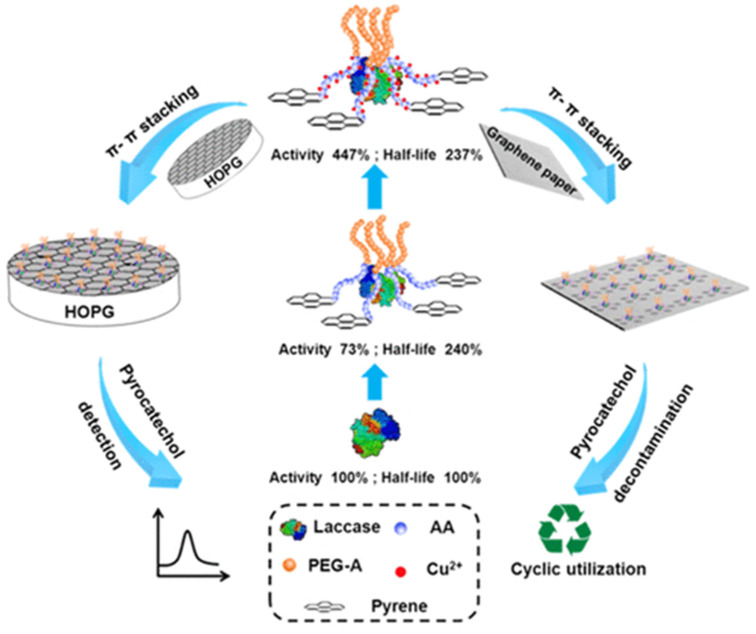
Schematic illustration for immobilization of laccase by the Cu^2+^/PAA/PPEGA matrix for the detection of pyrocatechol. Reprinted with permission from ref. [81]. Copyright 2017 American Chemical Society.

The accurate and efficient detection of small organic molecules has aroused great interest due to their relevance in diverse fields. Monoliths, capillary, poly(ionic liquid), magnetic polymers, and molecularly imprinted materials have been prepared through RAFT polymerization for the separation and detection of small organic molecules by liquid chromatography, capillary electrochromatography, mass spectrometry, and so on [58,59,60,61,62,63,64,65,66,67,68,69,70,71,72,73,74,75,76]. In contrast to these techniques, electrochemical sensors are considered auspicious tools to determine small organic molecules relevant to health diagnostics and environmental monitoring due to their advantages of cost-efficiency, short response time, ease of use, high sensitivity, and ease of miniaturization. Polymers and polymeric nanomaterials such as carbon nanotubes, graphene, metal nanoparticles, and magnetic beads have been synthesized by RAFT polymerization and used as electrode materials for sensing applications. The RAFT polymerization technique can facilitate the preparation of polymers as the sensor materials or carriers of enzymes for direct or enzymatic detection of small organic molecules (Table 3) [77,78,79,80]. For example, Singh et al. reported the electrochemical detection of dopamine using nanomicelle block copolymers of poly(*N*-vinylcarbazole) [PNVCz] incorporated with three distinct sulfonic acid polymer derivatives [77], including poly(*N*-vinylcarbazole)-*b*-poly(2-methyl-2-propene-1-sulfonic acid) [PNVCz-*b*-PMPSA], poly(*N*-vinylcarbazole)-*b*-poly(2-acrylamido-2-methyl-1-propanesulfonic acid) [PNVCz-*b*-PAMPS], and poly(*N*-vinylcarbazole)-*b*-poly(styrenesulfonic acid) [PNVCz-*b*-PSSA]. The polymers were synthesized by the RAFT polymerization technique with (2-ethyl isobutyrate)-(O-ethyl xanthate) as the CTA. Their self-organization and ion-exchange properties facilitated the detection of dopamine even in the presence of ascorbic acid. Rajarathinam et al. developed an electroenzymatic biosensor for glutamate detection using copolymer poly(2-dimethylaminoethyl methacrylate-styrene) to non-covalently functionalize reduced graphene oxide (rGO) (DS–rGO) and immobilize glutamate oxidase (GluOx) [79]. The enzyme nanosheets (ENs) were drop-coated over Prussian blue (PB)-electrodeposited screen-printed carbon electrode (SPCE) for real-time monitoring of glutamate release from primary cortical neurons. Chen et al. reported an electrochemical biosensor for pyrocatechol detection by using a Cu^2+^-adsorbed pyrene-terminated RAFT block copolymer [poly(acrylic acid)/poly(poly (ethylene glycol) acrylate)] (PAA/PPEGA) to immobilize laccase (Figure 7) [81]. In this work, PAA was used as a supporting matrix to immobilize enzyme bioactivity inducer Cu^2+^, and PPEGA served as a modifier to improve enzyme stability. The modified laccase could be attached onto highly oriented pyrolytic graphite (HOPG) and graphene paper through π−π stacking interaction between the pyrene moiety of PAA/PPEGA and the π-conjugated graphene-like surface. The modification method led to the enhancement of laccase catalytic bioactivity and stability to 447% and 237%, respectively. The electroenzymatic biosensor allowed for the detection of pyrocatechol at the concentration down to 50 nM.

**Figure 8:**
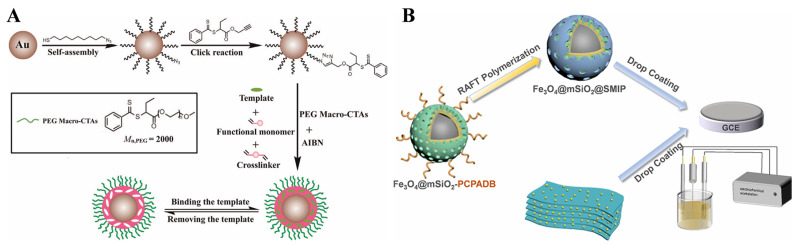
(**A**) Preparation of surface-imprinted polymer-coated AuNPs grafted with a water-compatible external layer by RAFTPP. Reprinted with permission from ref. [83]. Copyright 2014 Elsevier B.V. (**B**) Schematic illustration for the preparation of an imprinted polymer-based electrochemical sensor. Reprinted with permission from ref. [91]. Copyright 2025 American Chemical Society.

The molecular imprinting technique is an emerging and exploring method used to prepare sensing materials with improved target recognition capacity [82]. Molecular imprinting on a nano-surface can form molecularly imprinted polymers (MIPs) with high target-binding ability and mass transfer rate. MIPs-modified nanomaterials prepared by the RAFT polymerization technique have been used for sensing different small molecules, including AuNPs, carbon nanotubes, and graphene-based nanocomposites [83,84,85,86,87,88,89,90,91,92,93,94]. For example, Zhao et al. prepared fenitrothion-imprinted polymer-coated AuNPs by click chemistry and RAFT precipitation polymerization (RAFTPP) (Figure 8A) [83]. The RAFT CTAs were prepared on the nanoparticle surface by click chemistry. Fenitrothion-imprinted polymers with hydrophilic polyethylene glycol (PEG) brushes were then synthesized on the CTAs-modified AuNPs by RAFTPP. The MIPs could selectively recognize fenitrothion and achieved the electrochemical determination in the linear range of 0.01~5 μM. Mesoporous materials have the advantages of regular ordered pore structure and extremely high specific surface area and superior chemical and thermal stability, providing numerous template imprinted sites. Shao et al. reported an electrochemical biosensor for tetrabromobisphenol A (TBBPA) detection by modifying the electrode with magnetic surface MIP (SMIP) and MXene/Au nanocomposite (Figure 8B) [91]. The magnetic SMIP was prepared by SI-RAFT polymerization on the magnetic mesoporous nanosilica (Fe_3_O_4_@mSiO_2_) surface. The MXene/Au nanocomposite enhanced the electrochemical signal by synergistic effect. The modified electrode was used to determine TBBPA in water samples with the recovery changing in the range of 96~104%.

**Figure 9:**
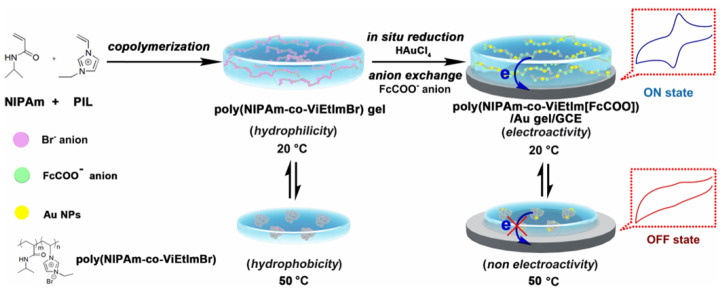
Schematic illustration for the fabrication of poly(NIPAm-*co*-ViEtIm[FcCOO])/Au gel for thermally responsive electrochemical response. Reprinted with permission from ref. [95]. Copyright 2023 American Chemical Society.

Poly(N-isopropyl acrylamide) (PNIPAm)-based thermally responsive gel can be used to modify sensing electrodes in response to temperature change. Polymer ionic liquid (PIL) is a solution system consisting of a polymer backbone and an ionic liquid with a continuous repeating unit structure. Because of its polymerizability, PIL has been combined with PNIPAm gel through copolymerization. Xia et al. prepared a thermally responsive electroactive poly(*N*-isopropyl acrylamide-*co*-ViEtIm[ferrocenecarboxylate anion])/Au named poly(*N*IPAm-*co*-ViEtIm[FcCOO])/Au gel (Figure 9) [95]. The gel was composed of copolymer poly(*N*IPAm-*co*-ViEtImBr), ferrocenecarboxylate anion (FcCOO^−^), and AuNPs to achieve thermally responsive redox activity and electrocatalytic activity. The PNIPAm-based gel was synthesized by RAFT polymerization with ionic liquid and NIPAm monomers. FcCOO^−^ anions were immobilized on the gel by an anion-exchange reaction. AuNPs generated by in situ reduction of HAuCl_4_ precursors were attached by electrostatic interactions. The thermally responsive gel could reveal or seal electroactive FcCOO^−^ by the conformational change in PNIPAm segments at different temperatures, thus switching its electrocatalytic performance toward ascorbic acid oxidation.

**Figure 10:**
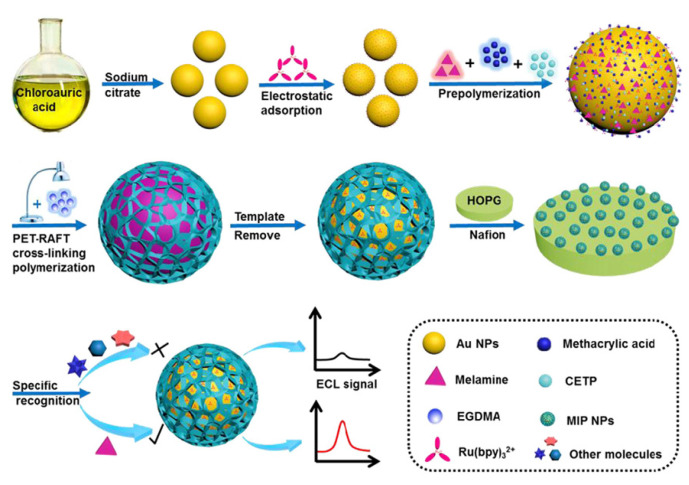
Schematic illustration for the preparation of Ru(bpy)_3_^2+^-immobilized AuNPs and melamine-MIPs used for ECL sensing of melamine. Reprinted with permission from ref. [99]. Copyright 2019 Elsevier B.V.

The electrochemiluminescence (ECL) sensing technique integrates the advantages of electrochemistry and chemiluminescence. It allows the signal to be turned on and off in a spatially localized manner and exhibits high sensitivity due to the extremely low background signal [96,97]. Liu’s group developed an ECL biosensor based on ruthenium (Ru)-complex (Ru(bpy)_3_^2+^)-catalyzed RAFT photopolymerization for the synthesis of pyrene-functionalized poly(sodium *p*-styrene sulfonate) (PSS) (Figure 10) [98]. The Ru/PSS-modified HOPG electrode was used for tripropylamine detection based on its reaction with Ru(bpy)_3_^2+^ on the electrode surface. Furthermore, Liu’s group reported the ECL detection of melamine by the combination of molecularly imprinted technique and RAFT polymerization [99]. In this work, Ru(bpy)_3_^2+^ served as a catalyst for PET-RAFT polymerization and as an ECL probe for signal readout. Specifically, poly(methacrylic acid) (PMAA) and cross-linked PMAA were synthesized through Ru(bpy)_3_^2+^-catalyzed PET-RAFT polymerization under visible light illumination. Then, the negatively charged AuNPs were modified with Ru(bpy)_3_^2+^ by electrostatic incorporation. Melamine-templated MIPs were formed on the surface of AuNPs through PET-RAFT polymerization. The AuNPs-MIPs were coated on the HOPG electrode surface with the assistance of Nafion. The modified electrode could be used for the detection of melamine with a detection limit of 0.1 pM.

**Table 3:** Analytical performances of RAFT-based electrode materials for the detection of small molecules.

Material	Analyte	Linear Range	Detection Limit	Ref.
Block copolymers	Dopamine	5 × 10^−2^–1.5 mM	0.05 mM	[77]
Triblock copolymers	Syringic acid	1.5–15 µg/mL	0.44 µg/mL	[78]
GluOxENs/PB	Glutamate	3.25–250 μM	0.96 μM	[79]
Laccase/polymers	Pyrocatechol	5 × 10^−5^–1 mM	50 nM	[81]
MWCNTs@ZnO/PMAEFc	Aspartame	10^−3^–10 nM	1.35 nM	[80]
AuNPs@MIPs	Fenitrothion	10^−2^–5 μM	8 nM	[83]
MWCNTs@MIPs	Brucine	0.6–5.0 μM	2 nM	[84]
MWCNTs@MIPs	Imidacloprid	0.2–24 μM	0.08 μM	[85]
Fe_3_O_4_-MIP@rGO	17β-Estradiol	5 × 10^−2^–10 μM	0.819 nM	[86]
GO@MIPs	Glucose	1.5–1500 μM	5.88 μM	[87]
GO@MIPs	Methylparathion	0.2–200 ng/mL	4.25 ng/mL	[88]
Au@Fe_3_O_4_@rGO-MIPs	Ractopamine	2–100 nM	0.02 nM	[89]
MSMIP/rGO	Tetracycline	1.6–88 nM	0.916 nM	[90]
Fe_3_O_4_@mSiO_2_@SMIP	TBBPA	1–4500 nM	0.83 nM	[91]
Polymer/AuNPs gel	Ascorbic acid	0.1–25.8 mM	92.9 μM	[95]
Ru@pyrene-PSS	Tripropylamine	1–5000 μM	0.1 nM	[98]
Ru@AuNPs@MIPs	Melamine	5 × 10^–7^–5 μM	0.1 pM	[99]

Abbreviations: GluOxENs, glutamate oxidase-based enzyme nanosheets; PB, Prussian blue; MWCNTs, multi-walled carbon nanotubes; PMAEFc, poly(2-methacryloyloxyethyl ferrocenecarboxylate); AuNPs, gold nanoparticles; MIPs, molecularly imprinted polymers; rGO, reduced graphene oxide; MSMIP, magnetic molecularly imprinted polymer; SMIP, surface molecularly imprinted polymer; TBBPA, tetrabromobisphenol A; PSS, poly(sodium *p*-styrene sulfonate).

#### 4.2.2. Optical Sensing of Small Molecules

**Figure 11:**
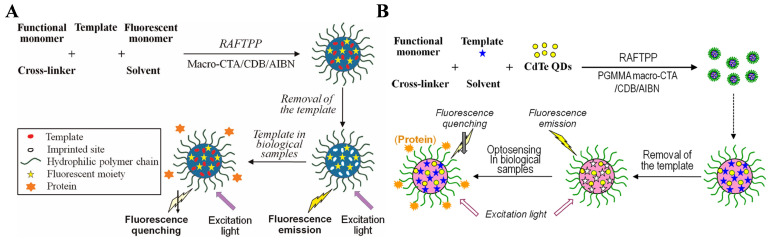
(**A**) Schematic illustration for the synthesis of hydrophilic and fluorescent MIP nanoparticles for drug optosensing. Reprinted with permission from ref. [105]. Copyright 2015 Elsevier B.V. (**B**) Schematic illustration for the one-pot synthesis of QD-labeled hydrophilic FA-MIP nanoparticles for FA optosensing. Reprinted with permission from ref. [106]. Copyright 2016 Elsevier B.V.

Polymer-based optical sensors represent a revolutionary advancement in biomedical diagnosis and monitoring due to their unique flexibility, biocompatibility, and selective responsiveness. RAFT polymerization has emerged as one of the most widely used techniques for the fabrication of multiblock copolymers and complex polymeric architectures [100]. Optical sensors with the signal tags and recognition moieties in the side chains of RAFT polymers have been developed for the detection of small organic molecules (Table 4). The polymer materials are usually prepared by RAFT polymerization of multifunctional monomers in solution or on the surface of solid supports. In addition, macromolecules and natural polymers can be grafted onto the polymeric backbones to improve water solubility and biocompatibility of copolymers [101,102,103,104]. For instance, Zhang’s group synthesized tetracycline-templated fluorescent MIP nanoparticles with hydrophilic polymer brushes by poly(2-hydroxyethyl methacrylate) (PHEMA) macromolecular chain transfer agent (micro-CTA)-mediated RAFTPP (Figure 11A) [105]. MAA, ethylene glycol dimethacrylate (EGDMA), cumyl dithiobenzoate (CDB), and azobisisobutyronitrile (AIBN) were used as the functional monomer, cross-linker, RAFT agent, and free radical initiator, respectively. Binding of tetracycline to the fluorescent MIP nanoparticles caused significant fluorescence quenching by a charge transfer mechanism. At the same time, Liu’s group synthesized CdTe quantum dots (QDs)-labeled hydrophilic MIP nanoparticles for fluorescent sensing of folic acid (FA) (Figure 11B) [106]. Hydrophilic micro-CTA-mediated RAFTPP was used to imprint FA on the surface of CdTe QDs. The resulting FA-imprinted polymer nanoparticles could selectively determine FA by electron transfer mechanism. The two fluorescent MIP-based sensors exhibited promising applications for clinical diagnostics and food analysis.

**Figure 12:**
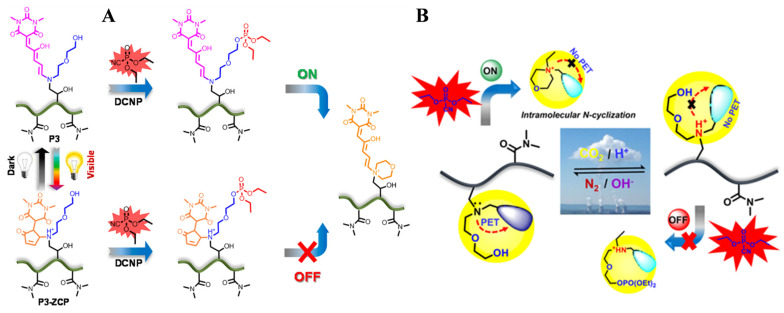
(**A**) Schematic representation of an on−off photoswitch for the colorimetric sensing of DCNP. Reprinted with permission from ref. [107]. Copyright 2016 American Chemical Society. (**B**) Schematic diagram of CO_2_/pH-responsive polymeric probe for the turn-on fluorescent detection of nerve agent mimics with tunable sensitivity. Reprinted with permission from ref. [108]. Copyright 2017 American Chemical Society.

In addition, Lee et al. designed a polymeric probe by RAFT polymerization of glycidyl methacrylate (GMA) and DMA monomers for colorimetric on–off detection of nerve agent mimics diethyl cyanophosphate (DCNP) (Figure 12A) [107]. The probe was derived from a visible light responsive donor–acceptor Stenhouse adduct (DASA). The yielded poly(glycidyl methacrylate-*co*-dimethylacrylamide) [p(GMA-*co*-DMA)] (P1) reacted with 2-(2-aminoethoxy)ethanol to form 1-((2-(2-hydroxyethoxy)ethyl)amino)-3-methoxypropan-2-ol (P2). The subsequent reaction between the secondary amine of P2 with 5-(furan-2-ylmethylene)-1,3-dimethylpyrimidine-2,4,6(1*H*,3*H*,5*H*)-trione yielded P3 with DASA derivatives. The color of the P3 solution/film turned from purple to colorless in the presence of DCNP, which can be attributed to the formation of morpholino cations caused by DCNP-promoted intramolecular N-alkylation. The probe P3 also exhibited good photochromic performance under visible light irradiation. The formation of zwitterionic cyclopentenone by visible light irradiation limited the DCNP-induced intramolecular N-alkylation. In addition, the p(GMA-*co*-DMA) could react with 2-(2-((pyren-1-ylmethyl)-amino)-ethoxy)-ethanol to yield a pyrene-based fluorescent polymeric probe for the tunable detection of DCNP (Figure 12B) [108]. The sensor was mediated by CO_2_/N_2_ gas or solution pH. The CO_2_/pH controllable fluorescence detection of DCNP was realized at high but not low pH. These two works provided new insights for the design of photoresponsive sensors for the detection of nerve agent mimics.

**Figure 13:**
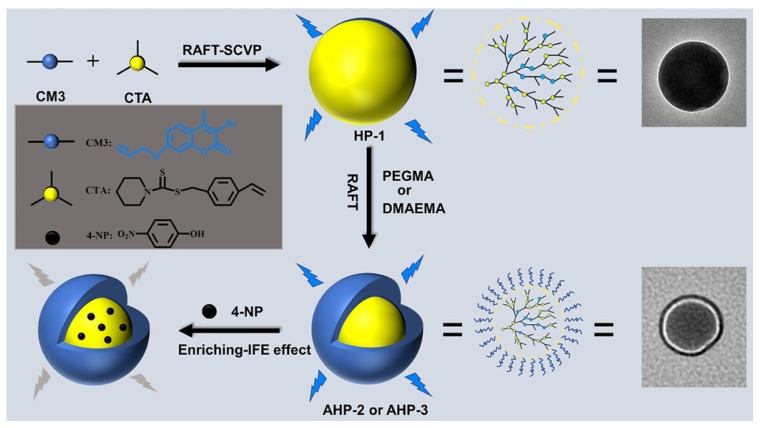
Schematic illustration of the synthesis of amphiphilic hyperbranched polymers for 4-NP detection. Reprinted with permission from ref. [109]. Copyright 2023 American Chemical Society.

Amphiphilic hyperbranched polymers (AHBPs) have the properties of high solubility, low viscosity, and multiple functional groups. Zhang et al. synthesized two amphiphilic hyperbranched fluorescent probes for the detection of 4-nitrophenol (4-NP) (Figure 13) [109]. The CTA with a styrene unit was synthesized and polymerized with a coumarin derivative CM3 via RAFT-self-condensing vinyl polymerization (RAFT-SCVP) to form hyperbranched polymer HP-1. Amphiphilic hyperbranched fluorescent probes AHP-2 and AHP-3 were then prepared with HP-1 as a macromolecular CTA for RAFT polymerization of PEGMA and DMAEMA monomers. The prepared AHP-2 and AHP-3 were used as fluorescent probes for 4-NP detection by “enriching inner filter effect (IFE)” with a detection limit of 0.59 μM.

**Figure 14:**
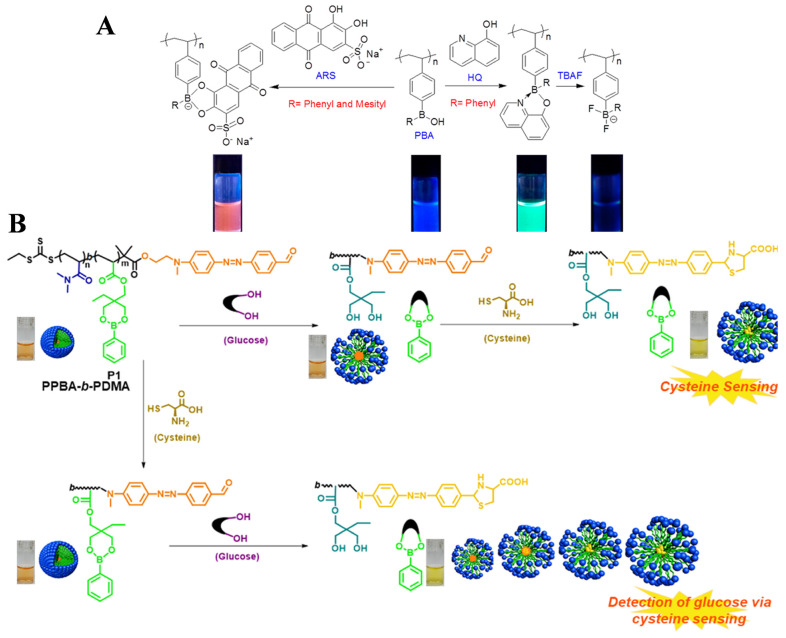
(**A**) Schematic illustration of the proposed mechanism of structural response of substituted PBA to diol compound ARS, electron-rich compound HQ, and fluoride ion under irradiation with a UV lamp at 365 nm. Reprinted with permission from ref. [112]. Copyright 2017 American Chemical Society. (**B**) Schematic representation for colorimetric detection of cysteine and glucose and their dependence on each other. Reprinted with permission from ref. [113]. Copyright 2021 American Chemical Society.

Boronic acid-containing polymers exhibit promising applications for sensing sugars and glycoproteins, electronic devices, chemical catalysis, and biomedical applications (e.g., self-healing materials, therapeutic agents, and self-regulated drug delivery systems) due to their unique reactivity and stimuli-responsive behavior [110,111]. Wan et al. synthesized boronic acid-containing polymers by the RAFT polymerization technique [112]. The electron-deficient boronic acid monomers with different substituents were synthesized through the reaction of Grignard reagent with trimethoxyborane. The thermoresponsive property of the polymers was dependent upon the steric hindrance of the boronic acid substituent (Figure 14A). The binding affinity of such polymers with Alizarin Red S (ARS), 8-hydroxyquinoline (HQ), and fluoride ion decreased with the increase in steric hindrance of substituent. The polymer with a phenyl substituent can be used for HQ detection at the ppm level due to the strong dative N→B bond and strongly luminescent boron quinolate. In addition, Sharma et al. synthesized a block copolymer probe [PPBA-*b*-PDMA (P1)] by RAFT polymerization of (2-phenylboronic esters-1,3-dioxane-5-ethyl) methyl acrylate and DMA monomers (Figure 14B). A cysteine-detectable aldehyde-functionalized azobenzene was used as the chain transfer agent. The probe could self-assemble into a micelle in a neutral pH aqueous solution. Cysteine did not induce the colorimetric change in the micellar solution because it could not reach the hidden aldehyde in the micelle. The addition of glucose made the micelle swollen due to the change in their hydrophilic behavior induced by the conversion of boronic ester into boronic acid. The exposed aldehyde group in the azo receptor could react with cysteine to cause the color change. This probe showed a dual-response property for cysteine and glucose sensing.

**Table 4:** Analytical performances of RAFT-based materials for optical detection of small molecules.

Method/Material	Analyte	Linear Range	Detection Limit	Ref.
Fluorescence/Cellulose	4-nitrophenol	10–10 μM	0.46 μM	[101]
Fluorescence/Chitosan	4-nitrophenol	0–10 μM	54 nM	[102]
Fluorescence/Polyampholyte	CS_2_	0–124 mM	123 µM	[103]
Fluorescence/MIP NPs	tetracycline	0.5–20 μM	0.26 μM	[105]
Fluorescence/MIP-QDs	folic acid	5 × 10^−2^–10 μM	25 nM	[106]
Color/Polymer	DCNP	0.5–6 mM	1 mM	[107]
Fluorescence/Polymer	DCNP	0–0.6 mM	0.1 mM	[108]
Fluorescence/Polymer	4 nitrophenol	0.1–18 mM	0.59 μM	[109]
Fluorescence/Copolymer	TNP	0–80 ppm	19 ppm	[114]
Fluorescence/GO@ZnS NPs	TNP	0.2–16 nM	4.4 nM	[115]
Fluorescence/MIP-GO	histamine	0.1–1000 M	25 nM	[116]
Fluorescence/Alq3-GO	TNP	0.12–2 nM	2.38 nM	[117]
Fluorescence/PBA polymer	HQ	5 × 10^−2^–3 ppm	–	[112]
color /PBA polymer	glucose	3–30 mM	3 mM	[113]
SERS/Polymer	aflatoxin B1	–	10 ppb	[118]

Abbreviations: MIP NPs, molecularly imprinted polymer nanoparticles; QDs, quantum dots; DCNP, diethyl cyanophosphate; TNP, 2,4,6-trinitrotoluene; GO, graphene oxide; Alq_3_, tris(8-hydroxyquinoline)aluminum; PBA, boronic acid; HQ, 8-hydroxyquinoline; SERS, surface-enhanced Raman scattering.

### 4.3. Polymeric Materials Prepared by RAFT Polymerization for Bioimaging

**Figure 15:**
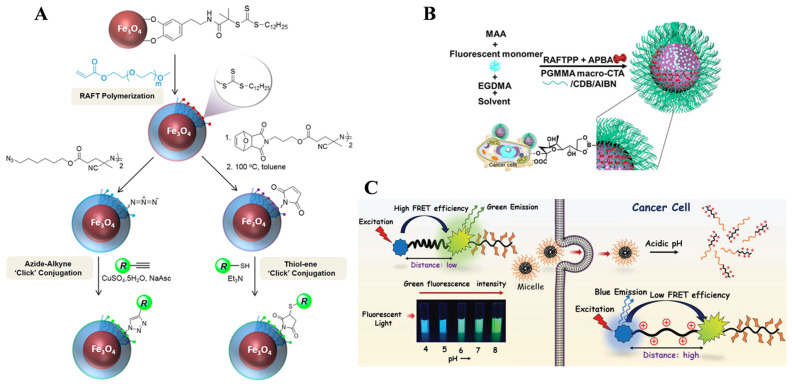
(**A**) Schematic illustration for the synthesis and modular post modification of polymer-coated MNPs. Reprinted with permission from ref. [130]. Copyright 2016 American Chemical Society. (**B**) Schematic illustration for the synthesis of the fluorescent nanoparticles. Reprinted with permission from ref. [131]. Copyright 2020 American Chemical Society. (**C**) Schematic representation of cellular uptake of PDPAEMA-*b*-FA-*b*-PMAG_AMC and pH-responsive FRET activity at different pH levels. Reprinted with permission from ref. [132]. Copyright 2022 Wiley-VCH.

RAFT polymerization provides unprecedented degrees of freedom for synthesizing water-soluble or amphiphilic structures with precise sizes and appropriate functionalities for delivery of diagnostic and therapeutic agents [119,120,121,122,123,124,125,126,127,128]. It has been used to produce block copolymer micelles, vesicles, star-shaped polymers, nanoparticles, and capsules as potential drug carriers and polymer–drug conjugates [129]. The applications of RAFT polymers in bioimaging are receiving increasing attention. For example, Oz et al. prepared polymer brush-coated magnetic nanoparticles (MNPs) by RAFT polymerization with different “clickable” groups (Figure 15A) [130]. First, a trithiocarbonate-terminated polymer brush with a catechol group and initiator tag was attached to the MNPs based on metal–catechol interaction. Then, “clickable” functional groups of azide and maleimide were modified on the surface of MNPs for the conjugation of alkyne-containing and thiol-containing molecules through the alkyne–azide cycloaddition and thiol–ene conjugation, respectively. With this method, cell surface receptor targeting peptides and fluorescent dyes were immobilized on the MNPs for the selective recognition and imaging of cancer cells. Li et al. prepared hydrophilic poly(glyceryl monomethacrylate) (PGMMA) brush-functional nanoparticles by macromolecular chain-transfer-agent-adjusted RAFTPP (Figure 15B) [131]. Fluorescent hydroxyethyl anthrancene-9-carboxylate methacrylate (AnHEMA) was employed as the comonomer to modify the nanoparticles. Phenylboronic acid (PBA) tags were used to label the nanoparticles for the recognition of glycans on living cells. Modification of the hydrophilic PGMMA brushes significantly improved surface hydrophilicity and reduced nonspecific binding of nanoparticles. In contrast to other fluorescent nanoparticles, the PGMMA brush-grafted nanoparticles exhibited enhanced fluorescence quantum yield (∼7%), longer lifetime (∼2 ns), and higher fluorescence stability. Finally, cancer cells were discriminated from normal cells with PGMMA-functional fluorescent nanoprobes. In addition, Bhattacharya et al. synthesized a FRET-based glycopolymer fluorescent probe by RAFT polymerization for distinguishing tumor cells and normal cells (Figure 15C) [132]. The two FRET moieties of fluorescein FA and 7-amino-4-methylcoumarin (AMC) were linked with pH-responsive polymer PDPAEMA. The resulting glycopolymer could undergo reversible swelling/deswelling at acidic/neutral conditions and showed high stability, good water solubility, and excellent specificity to image cancer cells based on FRET change. The pH-responsive system can also be used for monitoring targeted drug delivery by the fluorescence change on the variation in pH.

**Figure 16:**
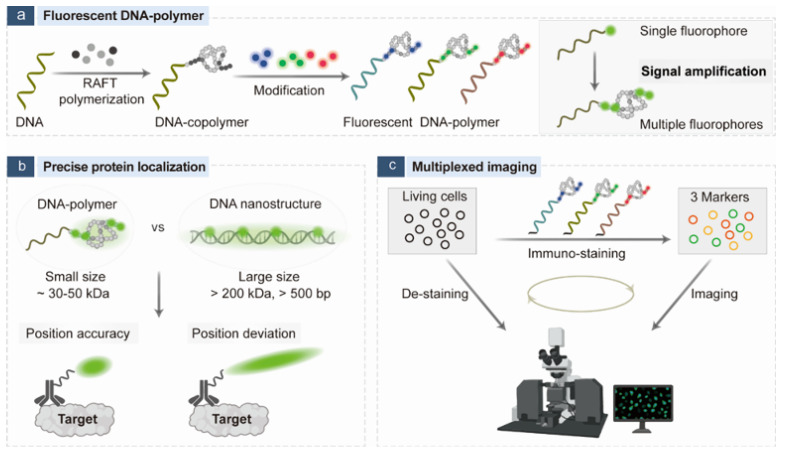
Schematic illustration for the synthesis of fluorescent DNA polymer through RAFT polymerization (**a**), which allows a single DNA strand to carry multiple fluorescent molecules at its end. (**b**) Schematic diagram of fluorescent DNA polymer for precision imaging applications. (**c**) Schematic diagram of fluorescent DNA polymer for multiplexed imaging applications of living cells. Reprinted with permission from ref. [133]. Copyright 2025 American Chemical Society.

Immunofluorescence imaging is a laboratory technique used in biology and medicine to visualize specific molecules within cells and tissues. This method employs specialized antibodies that are labeled with fluorophores to make the target structure visible under specific types of microscopes. It can provide insights into the location and distribution of biological components, helping to understand cellular processes and disease mechanisms. Fu et al. developed an immunofluorescence imaging method with fluorescent DNA polymers prepared through enzyme catalytic RAFT polymerization for signal amplification (Figure 16) [133]. DNA polymers—barcoded antibodies—were used for target recognition and fluorescence imaging. Multiplexed imaging of nine targets in live cells has been achieved. In contrast to other DNA-based immunofluorescence imaging methods, this approach can eliminate the requirement of complex DNA nanostructures and provide more precise protein localization.

**Figure 17:**
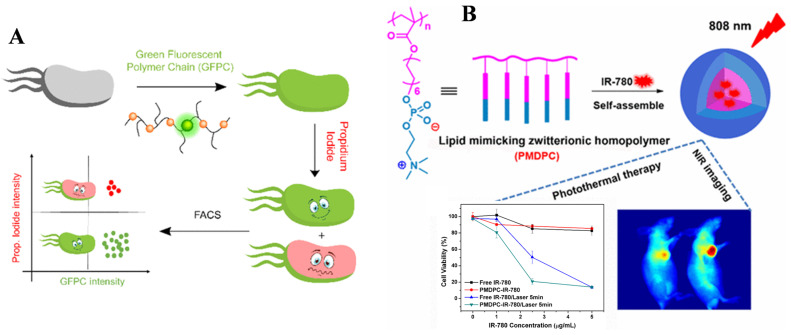
(**A**) Schematic illustration of fluorescent copolymers for bacterial bioimaging and viability detection. Reprinted with permission from ref. [136]. Copyright 2020 American Chemical Society. (**B**) Schematic illustration for the preparation of IR-780 encapsulated PMDPC micelles. Reprinted with permission from ref. [137]. Copyright 2016 American Chemical Society.

In addition to serving as bioimaging agents, polymers can also be simultaneously endowed with antibacterial or phototherapy properties for biomedical applications [134,135]. For instance, Sclavi’s group developed a nontoxic and water-soluble green fluorescent polymer chain (GFPC) by one-pot RAFT polymerization (Figure 17A) [136]. The polymer showing high photostability and biocompatibility could be used for multicolor bacterial bioimaging by binding to cytoplasm. Live and dead bacteria were distinguished and quantified by integrating this method with propidium iodide through flow cytometry. IR-780 iodide is a near-infrared (near-IR) fluorescent dye that can be used for both imaging and photothermal therapy. However, its biomedical application may be limited by its lipophilicity. To resolve this problem, Chen et al. synthesized a phospholipid-mimicking amphiphilic homopolymer, poly(12-(methacryloyloxy)dodecyl phosphorylcholine) (PMDPC), by RAFT polymerization (Figure 17B) [137]. The homopolymer can self-assemble into micelles for the encapsulation of IR-780. The resulting PMDPC-IR-780 micelle showed low cytotoxicity in the dark but exhibited remarkable photothermal cytotoxicity to pancreatic cancer cells under near-IR laser irradiation.

**Figure 18:**
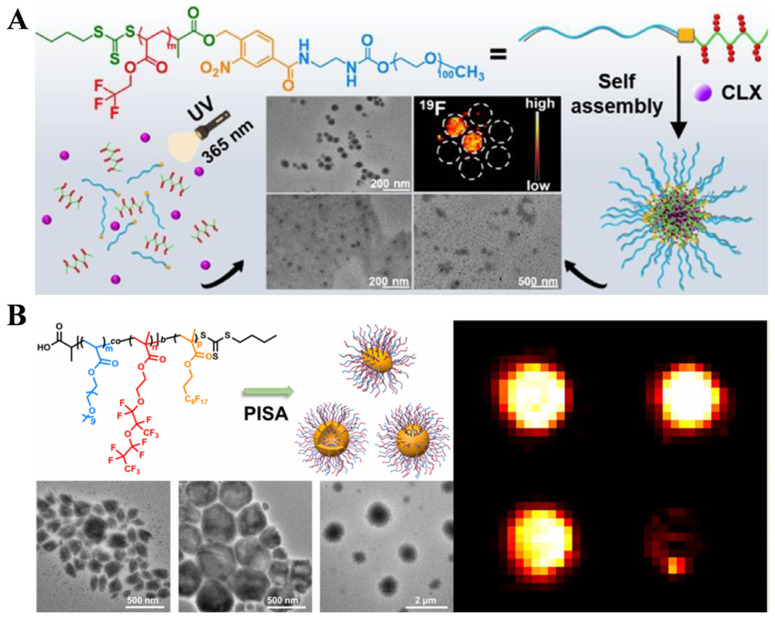
(**A**) Schematic illustration for the preparation of photo-responsive drug-loaded ^19^F MRI nanoprobes. Reprinted with permission from ref. [142]. Copyright 2023 American Chemical Society. (**B**) Schematic diagram for the synthesis of ^19^F MRI contrast agents by the RAFT-mediated self-assembly method. Reprinted with permission from ref. [143]. Copyright 2023 American Chemical Society.

Magnetic resonance imaging (MRI) is a non-invasive and ionizing radiation-free imaging technique for disease diagnosis [15,138,139]. Fluorine-19 magnetic resonance imaging (^19^F MRI) probes have received considerable research interest as theranostic agents [140,141]. However, the probes are limited by fluorine content or the poor ability of fluorinated tracers. Zhao’s group prepared a series of photo-responsive amphiphilic block copolymers by RAFT polymerization for ^19^F MRI (Figure 18A). The copolymers with different chain lengths consisted of hydrophilic poly(ethylene glycol) and ^19^F-containing hydrophobic poly(2,2,2-trifluoroethyl acrylate) (PTFEA). Photosensitive functional groups of *o*-nitrobenzyl oxygen were included in the copolymers to mediate their photolysis behavior upon ultraviolet irradiation. The polymeric nanoparticles exhibited good imaging signal and sufficient drug-loading efficiency (10%) as well as cumulative release (49%), showing promising theranostic applications for ^19^F MRI. In addition, Zhao’s group synthesized polymeric nanoparticles with different morphologies (e.g., ellipsoidal, spherical nanoparticles and vesicles) by RAFT polymerization of oligo(ethylene glycol) methyl ether acrylate and perfluoropolyether methacrylate monomers for ^19^F MRI (Figure 18B). All of the nanoparticles exhibited low or no cytotoxicity, showing great potential as ^19^F MRI agents for in vivo imaging applications.

## 5. Biosensors by the Signal Amplification of RAFT Polymerization Technique

### 5.1. Electrochemical Biosensors Based on RAFT Polymerization for Signal Amplification

Electrochemical biosensors have aroused great research interest in the fields from environmental monitoring to food safety and disease diagnosis. In order to improve the sensitivity of electrochemical biosensors for the detection of low-abundance targets, signal amplification is always necessary [144,145]. Nanomaterials have shown great potential in enhancing the sensitivity due to their excellent conductivity, high surface to volume ratio, and ease of functionalization. Polymers can be pre-prepared by the RAFT polymerization technique and used as the electrode materials for bioreceptor immobilization or as signal labels for signal readout [146,147]. More interestingly, surface-initiated RAFT (SI-RAFT) polymerization can be performed in situ to recruit a large number of electroactive tags at the electrode interface to amplify the signal. The activation methods for initiating radicals include thermal, electrochemical, photochemical, enzymatic, and mechanical stimulation. In this section, RAFT polymerization-based signal amplification for the design of electrochemical biosensors was discussed according to the difference in the methods for the formation of polymers.

#### 5.1.1. Thermal SI-RAFT Polymerization for Signal Amplification

**Figure 19:**
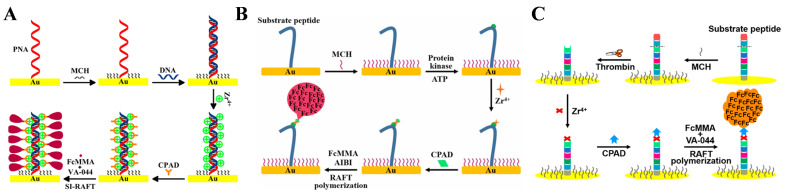
(**A**) Schematic illustration for the preparation of the SI-RAFT-polymerization-based electrochemical DNA biosensor. Reprinted with permission from ref. [148]. Copyright 2018 American Chemical Society. (**B**) Schematic illustration for the design principle of the peptide-based electrochemical biosensor. Reprinted with permission from ref. [154]. Copyright 2020 Elsevier B.V. (**C**) Schematic illustration for electrochemical biosensing of thrombin activity. Reprinted with permission from ref. [156]. Copyright 2020 American Chemical Society.

In RAFT polymerization, the initiating radicals can be supplied by different activation methods, including thermal, electrochemical, photochemical, enzymatic, and mechanical stimuli (Table 5). Thermal activation of radical initiators is the conventional means to initiate RAFT polymerization, which was first used for determining specific DNA sequences by surface-initiated polymer growth [25]. In recent years, the SI-RAFT polymerization technique has been successfully used to amplify the signals of electrochemical biosensors through the formation or deposition of redox-active polymer chains or metal nanoparticles [148,149,150,151,152,153,154,155,156,157,158,159,160]. In 2018, Hu et al. developed an SI-RAFT polymerization-based electrochemical biosensor for DNA detection with peptide nucleic acid (PNA) as the capture probe (Figure 19A) [148]. After the capture of target DNA, 4-cyano-4-(phenylcarbonothioylthio)pentanoic acid (CPAD) was attached onto the PNA−DNA duplexes through the carboxylate−Zr^4+^−phosphate interaction. With 2,2′-azobis[2-(2-imidazolin-2-yl)propane] dihydrochloride (VA-044) as the azo free-radical chain transfer agent, SI-RAFT polymerization of ferrocenylmethyl methacrylate (FcMMA) monomers was thermally initiated under a mild condition. Through SI-RAFT polymerization, numerous electroactive Fc tags were introduced on the electrode surface to achieve signal amplification and significantly improve the detection sensitivity. The method had the advantages of low cost and easy operation since it did not require the use of natural enzymes or nanomaterials for signal amplification. The carboxylate−Zr^4+^ interaction has also facilitated the design of electrochemical biosensors for monitoring peptide/protein phosphorylation and hydrolysis [154,155,156]. For example, after the phosphorylation of substrate peptide by protein kinase A (PKA), the chain transfer agent CPAD can be linked onto the phosphorylated site through the carboxylate−Zr^4+^−phosphate interaction (Figure 19B). Then, the SI-RAFT polymerization of FcMMA monomers was initiated in the presence of free radical initiators to produce an amplified electrochemical signal. If the substrate peptide was proteolytically cleaved by protease such as thrombin (Figure 19C), the exposed carboxyl group could be used to conjugate CPAD with Zr^4+^ as the linker via carboxylate−Zr^4+^−carboxylate interaction. The captured CPAD could initiate the polymerization of FcMMA monomers with VA-044 as the azo free-radical chain transfer agent.

**Figure 20:**
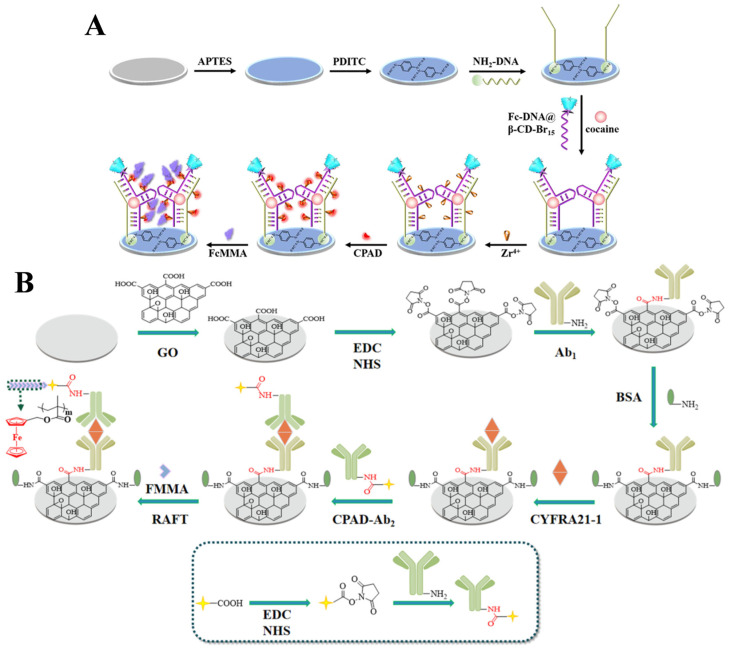
(**A**) Schematic description of a β-CD-Br15-based thermally mediated SI-RAFT electrochemical biosensor for cocaine sensitive detection. Reprinted with permission from ref. [157]. Copyright 2021 Elsevier B.V. (**B**) Schematic illustration of the electrochemical immunosensor by RAFT polymerization for the detection of CYFRA21-1. Reprinted with permission from ref. [158]. Copyright 2021 Elsevier B.V.

In addition to DNA, kinase, and protease biosensors, electrochemical aptasensors and immunosensors have also been developed by the signal amplification of SI-RAFT polymerization. For example, based on the carboxylate−Zr^4+^−phosphate interaction, Wang et al. suggested that cocaine captured by the DNA probe attached to the electrode surface could immobilize the ferrocene–DNA (Fc–DNA) signal probe by the specific aptamer–target binding (Figure 20A) [157]. Then, β-cyclodextrin β-CD-Br15 was attached to the electrode surface through the Fc–β-CD host–guest interaction. Through SI-RAFT polymerization with β-CD-Br15 as the initiator, FcMMA monomers were polymerized on the electrode surface with Zr^4+^ ions as the linkers to capture the chain transfer agent CPAD. Based on this detection principle, other targets can be determined by the signal amplification of SI-RAFT polymerization by changing the aptamer sequence. When the recognition antibody was labeled with chain transfer agent CPAD, an immunosensor can be developed based on the signal amplification of SI-RAFT polymerization of electroactive FcMMA [158]. For example, through the signal amplification of SI-RAFT polymerization, cytokeratin fragment antigen 21-1 (CYFRA21-1) has been determined with a linear range of 0.5 fg/mL~10 pg/mL and a detection limit of 0.14 fg/mL (Figure 20B).

#### 5.1.2. eRAFT Polymerization for Signal Amplification

**Figure 21:**
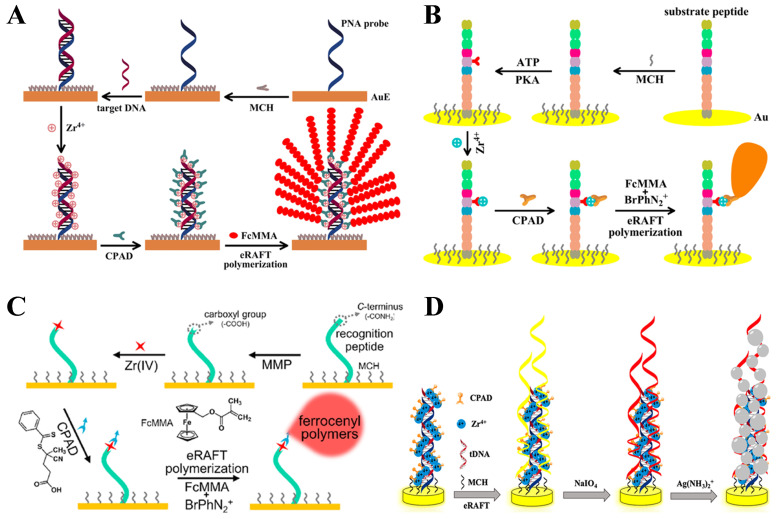
(**A**) Schematic illustration of the eRAFT polymerization-based electrochemical DNA biosensor. Reprinted with permission from ref. [161]. Copyright 2019 American Chemical Society. (**B**) Schematic illustration of the cleavage-based electrochemical PSA biosensor. Reprinted with permission from ref. [162]. Copyright 2019 American Chemical Society. (**C**) Principle of the ultrasensitive cleavage-based electrochemical MMP sensor. Reprinted with permission from ref. [163]. Copyright 2021 Elsevier B.V. (**D**) Schematic illustration of the electrochemical detection of DNA. Reprinted with permission from ref. [164]. Copyright 2019 American Chemical Society.

In the thermal SI-RAFT polymerization, the initiating radicals are generally produced by thermal decomposition of azo radical initiators at 50~70 °C. However, such an initiation temperature may induce the denaturation and/or deactivation of biomacromolecules such as DNA, enzymes, and target proteins. In addition, some initiators, including CPAD, are thermally unstable at the initiation temperature. For this view, the eRAFT polymerization technique has been developed and used for the design of electrochemical biosensors. Through the eRAFT polymerization, DNA, kinases, protease, and glycoproteins have been successfully determined by several groups [161,162,163]. Typically, Hu et al. reported an electrochemical DNA biosensor based on the signal amplification of eRAFT polymerization using a PNA probe as the recognition element (Figure 21A) [161]. The target DNA was labeled by chain transfer agent CPAD through the phosphate−Zr^4+^−carboxylate interaction. The eRAFT polymerization of FcMMA monomers was initiated by electrochemical reduction in aryl diazonium salts under a potentiostatic condition to generate free radicals. This brought numerous electroactive ferrocene (Fc) tags onto the electrode surface, resulting in an enhanced electrochemical signal. The eRAFT polymerization strategy has also been used for monitoring the activity of kinases and proteases [162,163]. After the phosphorylation (Figure 21B) or proteolysis (Figure 21C) of the substrate peptide, a chain transfer agent such as CPAD and α-bromophenylacetic acid (BPAA) could be anchored on the electrode surface through carboxylate−Zr^4+^−phosphate or carboxylate−Zr^4+^−carboxylate interaction. The eRAFT polymerization of FcMMA monomers was then initiated by aryl diazonium salts at a given potential to produce free radicals.

In addition to redox-active polymers, electro-inactive monomers can also be polymerized on the electrode surface by RAFT polymerization. The yielded polymers allowed for the attachment of electroactive species via electrostatic adsorption or the deposition of silver nanoparticles (AgNPs) through silver mirror reaction, thereby amplifying the electrochemical signals [150,151,152,153,164]. For example, the RAFT polymerization of N-acryloxysuccinimide can generate polymer chains that are able to recruit luminol for ECL detection of miRNA-21 [153]. Glycosyloxyethyl methacrylates (GEMA) can be assembled on the electrode surface by eRAFT to form sugar-containing polymers (Figure 21D) [164]. The *o*-hydroxyl group in the sugar skeleton was oxidized into an aldehyde group by NaIO_4_. Then, silver particles were in situ deposited on the electrode surface by the reduction of silver ions through silver mirror reaction. The formed silver particles could be electrochemically oxidized and determined by differential pulse voltammetry.

#### 5.1.3. Bioinspired eRAFT Polymerization for Signal Amplification

**Figure 22:**
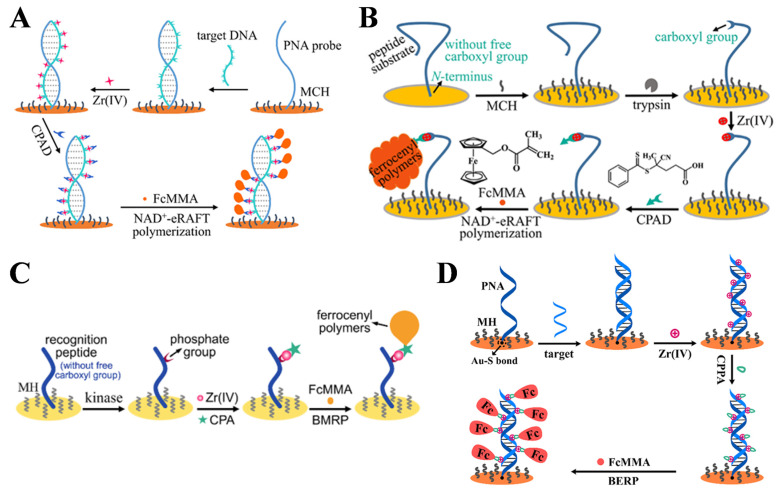
(**A**) Principle of the PNA-based electrochemical DNA biosensor. Reprinted with permission from ref. [165]. Copyright 2021 Elsevier B.V. (**B**) Principle of the cleavage-based electrochemical interrogation of trypsin activity through NAD^+^-eRAFT polymerization. Reprinted with permission from ref. [166]. Copyright 2021 American Chemical Society. (**C**) Schematic illustration of BERP-based electrochemical sensing of nucleic acids. Reprinted with permission from ref. [167]. Copyright 2018 American Chemical Society. (**D**) Principle of the proposed kinase sensor. Reprinted with permission from ref. [169]. Copyright 2021 American Chemical Society.

The method of electro-grafting of polymers through eRAFT polymerization for signal amplification is efficient, easy to use, low-cost, and highly compatible with biological systems. However, only a few examples of eRAFT polymerization have been reported since the method requires the use of a suitable redox mediator to shuttle electrons from the electrode interface to the RAFT agent. In addition, the electro-reduction in an exogenous radical source may react with the propagating radical to irreversibly terminate the growth of the polymer chain. With coenzyme as the mediator, bioinspired eRAFT polymerization (BERP)-assisted growth of polymers without the use of exogenous radical sources is attractive. Niu’s group developed several electrochemical biosensors through nicotinamide adenine dinucleotide (NAD^+^)-mediated eRAFT (NAD^+^-eRAFT) polymerization of FcMMA [165,166,167,168,169,170,171]. For example, they developed a PNA-based electrochemical biosensor for DNA detection based on the NAD^+^-eRAFT-mediated electro-grafting of polymers for signal amplification (Figure 22A) [165]. The chain transfer agent CPAD was attached onto the captured DNA through carboxylate−Zr^4+^−phosphate interaction. Then, electro-reduction in the NAD^+^-mediated fragmentation of CPAD produced initiating radicals, triggering the polymerization of FcMMA on the electrode surface and producing a high electrochemical signal. At the same time, a signal-on protease electrochemical biosensor with trypsin as an example was designed with carboxyl group-free peptide as the substrate (Figure 22B) [166]. After cleavage of the peptide by trypsin, chain transfer agent CPAD was linked to the exposed carboxyl group with Zr^4+^ as the linker. Grafting of ferrocenyl polymers through the NAD^+^-mediated eRAFT polymerization was achieved by potentiostatic reduction in coenzyme NAD^+^. Based on the same signal amplification principle, protein kinase PKA was determined with a carboxyl group-free peptide as the substrate (Figure 22C) [167]. After phosphorylation, CPAD was tethered to the phosphate group through the carboxylate-Zr^4+^-phosphate linkage, initiating the NADH-mediated eRAFT reaction under a mild condition. These works suggested that the coenzyme-mediated electro-grafting of polymers exhibited great promise as a signal amplification method for the design of electrochemical biosensors. For nicotinamide-based enzymatic oxidation−reduction reactions such as NAD^+^/NADH and NADP^+^/NADPH, the redox reaction usually takes place at the N substituted pyridinium ring. Niu’s group found that other nicotinamide derivatives with a pyridinium center, such as 1-methylnicotinamide (MNA), can serve as redox mediators to mediate the eRAFT polymerization (Figure 22D) [169]. Then, a DNA biosensor was developed using PNA as the recognition probe. The captured DNA was labeled by CPAD with Zr^4+^ as the linker. Electro-reduction in MNA caused the fragmentation of CPAD moieties into radical species, triggering the polymerization of ferrocenyl monomers and thereby recruiting plenty of electroactive Fc tags for signal amplification.

**Figure 23:**
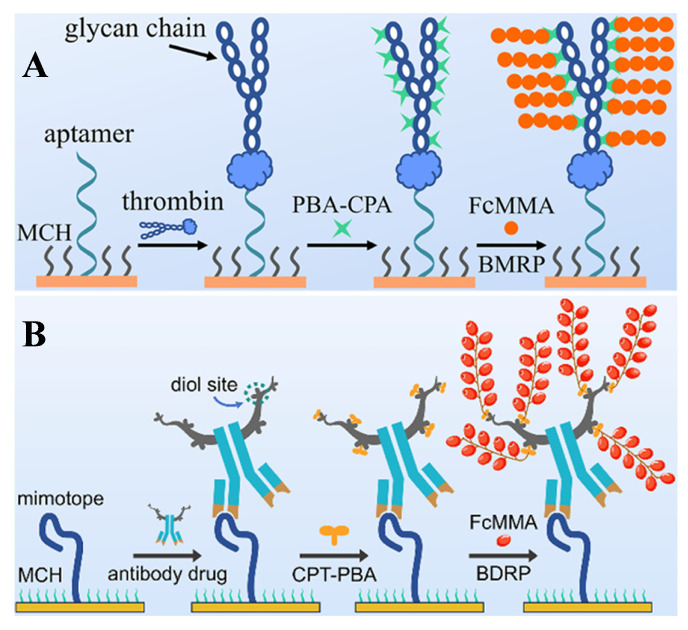
(**A**) Principle of the electrochemical thrombin aptasensor. Reprinted with permission from ref. [170]. Copyright 2023 American Chemical Society. (**B**) Principle of the BDRP-based electrochemical assay of antibody drugs. Reprinted with permission from ref. [171]. Copyright 2025 Elsevier B.V.

In the above examples for distinct RAFT polymerization methods, the chain transfer agent was generally linked to the target site, such as phosphate and carboxyl, with Zr^4+^ as the linker. Glycoproteins with saccharides in the glycan chains contain a large number of cis-1,2 or 1,3-diol moieties that can cross-link with boracic acid receptors. The boronate affinity-mediated molecular recognition and signal amplification have been exploited for the development of different biosensors [172]. Based on the target-synergized biologically mediated RAFT polymerization (*ts*BMRP), glycoproteins have been determined with aptamers as the recognition elements [170,171]. The captured glycoproteins, such as thrombin (Figure 23A) and antibody drug (Figure 23B), could be decorated by PBA-containing RAFT agents (PBA-CPA) through boronate affinity. Then, BMRP of FcMMA occurred on the electrode surface with NADH as the mediator. The in situ grafting of redox-active polymers achieved the signal amplification detection by monitoring the oxidation current of Fc tags.

#### 5.1.4. PET-RAFT Polymerization for Signal Amplification

**Figure 24:**
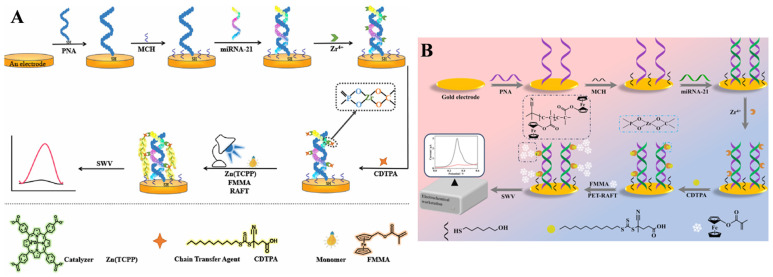
(**A**) Mechanism diagram of the PET-RAFT polymerization signal amplification electrochemical sensor for miRNA-21 detection. Reprinted with permission from ref. [173]. Copyright 2023 American Chemical Society. (**B**) Proposed mechanism of the biosensor based on PET-RAFT. Reprinted with permission from ref. [174]. Copyright 2024 Elsevier B.V.

PET-RAFT is a controllable polymerization technology that combines traditional RAFT polymerization and photooxidation-reduction catalytic reaction under visible/infrared light irradiation. The technique has the advantages of low energy consumption, mild reaction conditions, controllable speed, high oxidation resistance, catalyst diversity, and chemical selectivity. Photoactive Zn(TCPP) can activate thiocarbonyl sulfide compounds through electron transfer. Sun et al. developed an electrochemical biosensor for nucleic acid sensing based on Zn(TCPP)-mediated PET-RAFT polymerization for signal amplification (Figure 24A) [173]. The capture of target miRNA-21 by the PNA probe facilitated the attachment of chain transfer agent CDTPA on the electrode surface with Zr^4+^ as the linker. Zn(TCPP) activated CDTPA through electron transfer under blue light irradiation to initiate the PET-RAFT polymerization of FMMA monomers. In addition, Yu et al. reported an electrochemical biosensor for miRNA-21 detection based on PET-RAFT signal amplification in the co-existence of photocatalyst (erythrosin B) and tertiary amine co-catalyst (triethanolamine) (Figure 24B) [174]. Trithiocarbonate was activated by electron transfer under blue light (455~470 nm) irradiation, initiating the polymerization of FMMA monomers and realizing the signal amplification.

**Table 5:** Overview of electrochemical biosensors with the signal amplification of different RAFT methods.

RAFT Method	Analyte	Linear Range	Detection Limit	Ref.
SI-RAFT	DNA	10^−5^–10 pM	3.2 aM	[148]
	DNA	10^−2^–10 pM	1.51 aM	[149]
	DNA	10^−7^–1 nM	0.89 aM	[150]
	DNA	10^−6^–10 pM	0.487 aM	[151]
	DNA	10–10^6^ aM	5.6 aM	[152]
	miRNA-21	10^−5^–1 pM	0.21 aM	[153]
	PKA	0−140 mU/mL	1.05 mU/mL	[154]
	PKA	10^−7^–10^−2^ mU/mL	3.4 mU/mL	[155]
	thrombin	10−250 μU/mL	2.7 μU/mL	[156]
	cocaine	10^−2^–1000 ng/mL	3 pg/mL	[157]
	CYFRA21-1	0.5−10,000 fg/mL	0.14 fg/mL	[158]
	RdRP	5–500 aM	0.8 aM	[159]
	cTnI	10^−3^–1000 ng/mL	10.83 fg/mL	[160]
eRAFT	DNA	10^−5^–10 pM	4.1 aM	[161]
	PKA	0−140 mU/mL	1.02 mU/mL	[162]
	MMP-2	10^−3^–1 ng/mL	0.27 pg/mL	[163]
	DNA	10^−5^–1 pM	5.4 aM	[164]
Bioinspired eRAFT	DNA	10^−7^–0.1 nM	67 aM	[165]
	trypsin	25−175 μU/mL	18.2 μU/mL	[166]
	PKA	25−175 mU/mL	1.85 mU/mL	[167]
	DNA	10^−4^–10 pM	4.39 aM	[168]
	DNA	10^−6^–1 nM	0.58 fM	[169]
	thrombin	5×10^−2^–100 pM	35.3 fM	[170]
	RitMab	1–100 ng/mL	0.14 ng/mL	[171]
PET-RAFT	miRNA-21	10^−5^–100 pM	4.48 aM	[173]
	miRNA-21	10^−4^–100 pM	12.4 aM	[174]

Abbreviations: PKA, protein kinase A; CYFRA21-1, cytokeratin fragment antigen 21-1; RdRP, RNA-dependent RNA polymerase; cTnI, cardiac troponin I; MMP-2, matrix metallopeptidase 2; RitMab, antibody drug rituximab.

#### 5.1.5. Redox-Active Polymeric Materials for Signal Amplification

**Figure 25:**
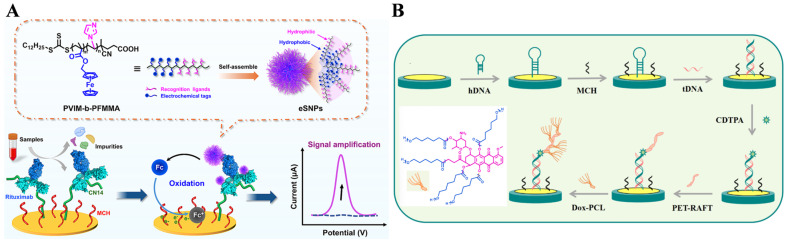
(**A**) Illustration of the preparation of eSNPs and their application in electrochemical detection of rituximab. Reprinted with permission from ref. [178]. Copyright 2024 Elsevier B.V. (**B**) Scheme diagram of electrochemical impedance spectroscopy biosensor for the detection of KRAS G12C mutations. Reprinted with permission from ref. [179]. Copyright 2025 Elsevier B.V.

In addition to the in situ polymerization methods, signal amplification can also be achieved by using pre-prepared polymers or polymeric materials as the signal labels of electrochemical biosensors, including redox-active polymers and polymer-modified nanoparticles [175,176,177,178,179,180]. For example, Chen et al. designed an electrochemical immunosensor for rituximab detection using amphiphilic block copolymer (PVIM-*b*-PFMMA) synthesized by the RAFT polymerization technique as the signal label (Figure 25A) [178]. The copolymer with 1-vinyl imidazole (VIM) as a recognition ligand for monoclonal antibody and ferrocenylmethyl methacrylate (FMMA) as a redox reporter was self-assembled into redox-active supramolecular nanoprobes (eSNPs). Rituximab was captured by the CN14 peptide-modified electrode. The eSNPs were attached to the electrode surface by the interaction between the VIM moiety and the constant region of rituximab. The accumulated FMMA redox tags resulted in remarkable signal amplification. This immunosensor can be used to determine rituximab in the serums collected from non-Hodgkin’s lymphoma (NHL) patients. In addition, Zhang et al. developed an electrochemical biosensor for monitoring KRAS G12C mutation by the signal amplification of PET-RAFT and ring-opening polymerization (ROP) (Figure 25B) [179]. The capture of target DNA (tDNA) by hairpin DNA (hDNA) anchored on the electrode surface facilitated the coupling of 4-cyano-4-[(dodecylsulfanylthiocarbonyl)sulfanyl]pentanoic acid (CDTPA) to initiate PET-RAFT polymerization of electro-active N-acryloxysuccinimide monomers. This provided a large number of junction sites for the conjugation of doxorubicin-polycaprolactone (Dox-PCL) synthesized by ROP, therefore leading to the signal change in electrochemical impedance spectroscopy.

### 5.2. Optical Biosensors Based on RAFT Polymerization for Signal Amplification

**Figure 26:**
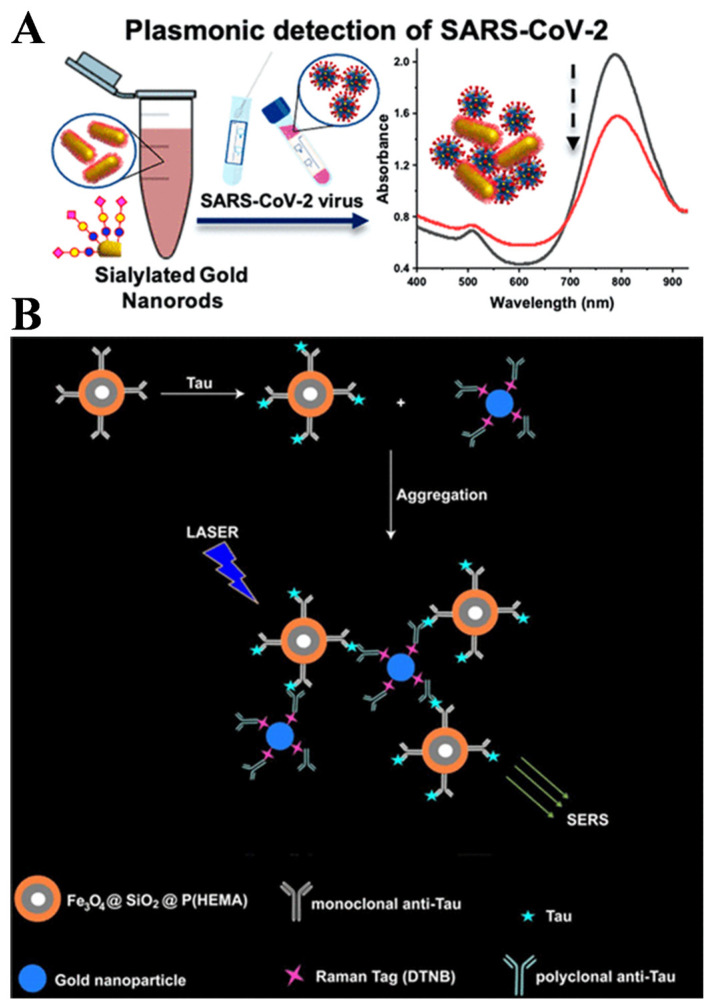
(**A**) Schematic illustration of gold nanorod-based plasmonic detection of SARS-CoV-2 spike protein. Reprinted with permission from ref. [187]. Copyright 2022 American Chemical Society. (**B**) Representative presentation of sandwich SERS assay. Reprinted with permission from ref. [188]. Copyright 2013 American Chemical Society.

Optical biosensors have enormous potential in clinical diagnostics, drug discovery, food control, and environmental monitoring due to their advantages, such as high sensitivity, robustness, reliability, and possibility to be integrated on a single chip. They mainly include fluorescence, colorimetry, surface plasmon resonance (SPR), surface-enhanced Raman scattering (SERS), and so on [181]. RAFT polymerization technique has been used to decorate solid surfaces for molecular immobilization and amplify the signals by the in situ formation of polymers [182,183,184,185]. Typically, early diagnosis of viral infection and disease control have been achieved based on RAFT polymerization and plasmonic phenomena, including aggregation-based colorimetric assays [186,187], surface-enhanced Raman scattering [188], propagating SPR [189,190], localized SPR [191,192], and surface-enhanced fluorescence [193]. For instance, Georgiou et al. reported an aggregation-based plasmonic colorimetric biosensor using glycosylated and polymer-stabilized gold nanorods to recognize SARS-CoV-2 spike protein (Figure 26A) [187]. In this work, telechelic polymer was synthesized by the RAFT polymerization technique for the capture and immobilization of 2,3-sialyllactose on gold nanorods. Glycan was introduced at the polymer termini for viral targeting and colloidal stability. SERS is a powerful analytical technique that can collect molecular spectral signals at the single-molecule level [194]. Zengin et al. designed an SERS-based magnetic homogeneous sandwich immunoassay platform for the detection of tau protein using AuNPs as the signal components (Figure 26B) [188]. The magnetic silica particles (Fe_3_O_4_@SiO_2_) coated with poly(2-hydroxyethyl methacrylate) by surface-mediated RAFT polymerization were functionalized with monoclonal anti-tau for the specific capture of tau protein. After magnetic separation, AuNPs modified with polyclonal anti-tau were attached and aggregated on the Fe_3_O_4_@SiO_2_ surface, allowing for the linear detection of tau at the concentration from 25 fM to 500 nM.

**Figure 27:**
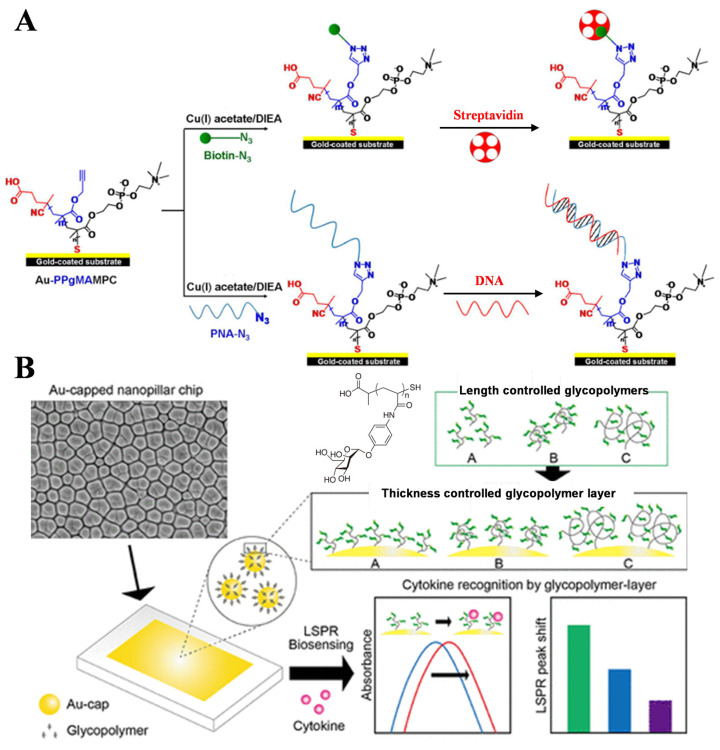
(**A**) Schematic illustration of immobilization of biotin-N3 and PNA-N3 on a PPgMAMPC-modified SPR chip for specific binding between conjugated probe and target molecule. Reprinted with permission from ref. [190]. Copyright 2016 American Chemical Society. (**B**) Schematic illustration of glycopolymer-immobilized Au-capped nanopillar for the detection of cytokine. Reprinted with permission from ref. [191]. Copyright 2021 American Chemical Society.

SPR is a widely available optical technique for monitoring the refractive index change in sensing layers. RAFT polymers can be used to decorate metal surfaces for suppressing nonspecific adsorption and immobilizing bioreceptors. Wiarachai et al. developed an SPR biosensor by using poly[(propargyl methacrylate)-ran-(2-methacryloyloxyethyl phosphoryl choline)] (PPgMAMPC) to decrease nonspecific adsorption (Figure 27A) [190]. The alkyne group propargyl methacrylate (PgMA) was introduced to the polymer as an active site for the conjugation of azide-containing molecules (e.g., biotin and PNA) by Cu-catalyzed click reaction. Then, SPR sensing platforms were developed based on the biotin−streptavidin interaction and PNA−DNA hybridization. LSPR is another potential candidate for plasmonic biosensing. Terada et al. developed an LSPR platform for the detection of cytokines using RAFT glycopolymer to capture the target (Figure 27B) [191]. The thickness of the biomolecular layer on the gold surface was controlled by changing the length of the glycopolymer chain. This work indicated the applicability of glycopolymer as a new material for cytokine recognition.

**Figure 28:**
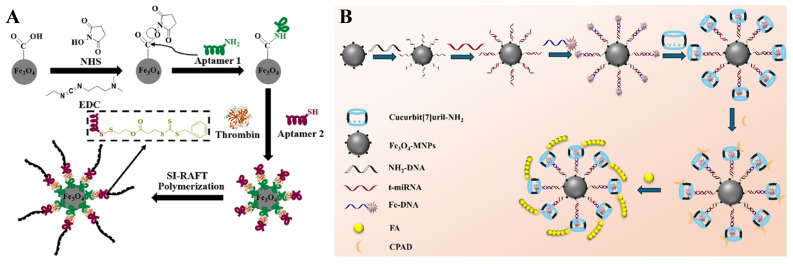
(**A**) Schematic illustration of a thrombin fluorescent sensor based on hemin-catalyzed SI-RAFT polymerization. Reprinted with permission from ref. [195]. Copyright 2023 Elsevier B.V. (**B**) Schematic illustration of fluorescent miRNA biosensor based on RAFT polymerization. Reprinted with permission from ref. [196]. Copyright 2026 Elsevier B.V.

In addition to being used as the interface modifiers, RAFT polymers can also be synthesized in situ by introducing a large number of signal tags on the sensing surface to develop optical biosensors. For example, Liu et al. reported a sandwich fluorescence aptasensor based on hemin-catalyzed SI-RAFT polymerization with thrombin as a model analyte (Figure 28A) [195]. The selective capture of thrombin by aptamer 1-modified Fe_3_O_4_ magnetic beads allowed for the attachment of thiol-modified aptamer 2. Through hemin-catalyzed SI-RAFT polymerization with FA as the monomer and acetylacetone (ACAC) as the initiator, the captured aptamer 2 was fluorescently labeled to achieve signal amplification. By monitoring the fluorescent signal of the FA tag, thrombin could be detected at a concentration down to 0.98 fM. In addition, Yao et al. reported a sandwich fluorescence biosensor for miRNA detection using Mo-based materials to initiate RAFT polymerization (Figure 28B) [196]. After the conjugation of Fc-labeled DNA (Fc-DNA) on the capture DNA-modified magnetic Fe_3_O_4_ with target miRNA (t-miRNA) as the linker, cucurbit[7]uril was anchored on the nanoparticle surface through the host–guest interaction between cucurbit[7]uril and Fc. This step was followed by the conjugation of CPAD through the amidation reaction and subsequent RAFT polymerization with FA as the monomer and MoO_4_^2−^ as the initiator to generate free radicals.

**Figure 29:**
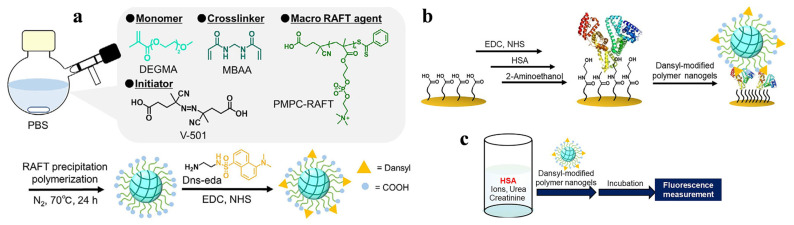
(**a**) Schematic of polymer nanogels with an albumin recognition/reporting interface synthesized through RAFT precipitation polymerization, (**b**) intermolecular interactions between dansyl-modified polymer nanogels and HSA-immobilized QCM sensor chip, and (**c**) fluorescence detection of urinary HSA by dansyl-modified polymer nanogels. Reprinted with permission from ref. [197]. Copyright 2025 Wiley-VCH.

Materials with recognizing and reporting dual functions are crucial for sensing applications. Kitayama et al. prepared polymer nanogels with the recognition/reporting dual function by RAFTPP of di(ethylene glycol) methyl ether methacrylate and N,N′-methylenebis(acrylamide) using a poly(2-methacryloyloxyethyl phosphorylcholine) (PMPC) macro-RAFT agent (Figure 29) [197]. Herein, PMPC bases play an important role in preventing nonspecific protein binding. After modification with dansyl groups, the nanogels were used for the recognition/reporting of proteins. The adsorption capacity of the dansyl-modified polymer nanogels for albumin protein was validated by quartz crystal microbalance (QCM) and polyacrylamide gel electrophoresis. Then, the polymer nanogels were used to report the adsorption of albumin from human (HSA), bovine (BSA), and porcine serum by monitoring the changes in fluorescence intensity and wavelength.

## 6. Challenges and Future Perspectives

The RAFT polymerization technique has shown great value in the preparation of sensing materials and design of various sensors due to its precise control over polymer structure and function. It provides critical technological support for the development of high-performance sensors. At present, significant advancements have been made in the design of sensors based on the RAFT polymerization technique. Functional polymers containing specific recognition groups (such as antigens, antibodies, MIPs, aptamers, etc.) can be designed and synthesized through RAFT polymerization, significantly improving the selectivity of sensors. In addition, the polymerization characteristics can enable signal amplification and improve detection sensitivity for different sensing fields such as biomolecule detection, environmental pollutant monitoring, and bioimaging. Despite these advancements, RAFT polymerization still faces some challenges that need to be addressed in sensing applications. For example, some RAFT polymerization-based sensors are prone to interference in complex real sample matrices, and their detection stability and anti-interference need to be improved. Most of the studies are still in the laboratory stage, with limited adaptability to practical field applications, and there is still a gap before commercialization. In addition, experimental conditions should be further optimized since residual CTAs during RAFT polymerization may affect the performance of sensors.

In the future, the development of RAFT polymerization in the sensing field can focus on the following directions: (1) design and synthesis of new functional polymers with multiple signal response units for developing sensors with multi-parameter detection capabilities, (2) optimization of sensing architecture and detection systems to improve the anti-interference ability and analytical accuracy in complex environments, and (3) integration of RAFT with other emerging technologies such as microfluidic chips, 3D printing, and smartphones to develop portable and miniaturized on-site detection sensors for commercial applications. With the continuous deepening of research, the RAFT polymerization technique is expected to play an increasingly important role in the field of sensors, providing strong support for the development of new sensing materials and detection technologies used for different scenarios.

## Figures and Tables

**Table 1:** A comparison of RAFT and ATRP [4].

Consideration	RAFT	ATRP
How does reversible deactivation occur?	Degenerate transfer: P_n_^•^ + CTA–P_m_ ⇌ CTA–P_n_ + P_m_^•^	Atom transfer: P_n_–X + activator ⇌ P_n_^•^ + deactivator
How are radicals generated?	Initiator_2_ → 2Initiator^•^	R–X + LCu^I^X ⇌ R^•^ + LCu^II^X_2_
Macroradical concentration [P_n_^•^]	Similar [P_n_^•^] to conventional steady-state radical polymerization. Termination is not curbed; chain transfer competes with both termination and propagation	Low [P_n_^•^] due to activation–deactivation equilibrium. Low rate of termination
Theoretical degree of polymerization (*DP*)	*DP*_RAFT_ = [Monomer]/[CTA]	*DP*_ATRP_ = [Monomer]/[R–X]
Obtaining narrow molecular weight distributions	As well as sufficient deactivation, CTA must be consumed early in the reaction: r_ct_ > r_propagation_	As well as sufficient deactivation, R–X must be consumed early in the reaction: r_initiation_ > r_propagation_
Ensuring predictable molecular weights	CTA must be fully consumed for theoretical and experimental molecular weights to agree	R–X must be fully consumed for theoretical and experimental molecular weights to agree
Maximizing end- group fidelity	The number of dead chains in conventional RAFT is determined by the number of initiators that have decomposed. Using low [Initiator_2_] gives higher end-group fidelity	End-group fidelity is enhanced by selecting a catalyst that favors deactivation over activation, lowering [P_n_^•^] and r_termination_
Ease of implementation	Simpler to implement, it uses fewer components (monomer, radical initiator, and CTA). The activity of the monomer must be matched with the CTA. Reactions are similar to conventional radical polymerization with added CTA	More components: monomer, ATRP initiator, activator, deactivator, and ligand. The activity of the monomer has to be matched with the activity of the initiator and ligand
Reaching high monomer conversions	Radical generation continues until all radical initiators are consumed. If 100% conversion is achieved but the initiator is still present, then further termination can occur	Radical generation continues to occur even at 100% conversion, potentially impacting end-group fidelity
pH tolerance	Typically not tolerant to basic environments due to degradation of CTAs at high pH	Few examples under acidic conditions due to protonation of N-donor ligands at low pH
Practical downsides	Polymers are often yellow or pink in color. CTAs often have a bad odor, and polymers can require purification to avoid this	Polymers are often blue/green to brown in color. Residual metal species may be problematic for some applications
End group	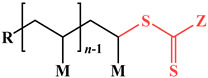	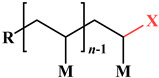

## Data Availability

Not applicable.
